# Biodiversity, environmental drivers, and sustainability of the global deep-sea sponge microbiome

**DOI:** 10.1038/s41467-022-32684-4

**Published:** 2022-09-02

**Authors:** Kathrin Busch, Beate M. Slaby, Wolfgang Bach, Antje Boetius, Ina Clefsen, Ana Colaço, Marie Creemers, Javier Cristobo, Luisa Federwisch, Andre Franke, Asimenia Gavriilidou, Andrea Hethke, Ellen Kenchington, Furu Mienis, Sadie Mills, Ana Riesgo, Pilar Ríos, Emyr Martyn Roberts, Detmer Sipkema, Lucía Pita, Peter J. Schupp, Joana Xavier, Hans Tore Rapp, Ute Hentschel

**Affiliations:** 1 GEOMAR Helmholtz Centre for Ocean Research Kiel, Düsternbrooker Weg 20, 24105 Kiel, Germany; 2 MARUM-Center for Marine Environmental Sciences and Department of Geosciences, University of Bremen, 28359 Bremen, Germany; 3 MPI-Max Planck Institute for Marine Microbiology, Celsiusstr. 1, 28359 Bremen, Germany; 4 AWI-Alfred Wegener Institute Helmholtz Centre for Polar and Marine Research, Am Handelshafen 12, 27570 Bremerhaven, Germany; 5 OKEANOS-Institute of Marine Research, University of the Açores, Rua Prof Frederico Machado, 9901-862 Horta, Portugal; 6 MARBEC, University of Montpellier, CNRS, IFREMER, IRD, Avenue Jean Monnet, CS 30171 - 34203, Sète, France; 7 IEO-CSIC-Spanish Oceanographic Institute, Oceanographic Centre Gijón, Avda. Principe de Asturias 70 bis, 33212 Gijón, Spain; 8 University of Bremen, Faculty 2 Biology/Chemistry, Leobener Str., 28359 Bremen, Germany; 9 IKMB-Institute of Clinical Molecular Biology, Rosalind-Franklin-Straße 12, 24105 Kiel, Germany; 10 Wageningen University, Laboratory of Microbiology, Stippeneng 4, 6708WE Wageningen, the Netherlands; 11 DFO-Department of Fisheries and Oceans, Bedford Institute of Oceanography, P.O. Box 1006, 1 Challenger Dr., B2Y 4A2 Dartmouth, NS Canada; 12 NIOZ-Royal Netherlands Institute for Sea Research, 1790 AB Den Burg Texel, the Netherlands; 13 NIWA-National Institute of Water and Atmospheric Research, 301 Evans Bay Parade Hataitai, Wellington, New Zealand; 14 MNCN-National Museum of Natural Sciences, Department of Biodiversity and Evolutionary Biology, c/José Gutiérrez Abascal 2, 28006 Madrid, Spain; 15 NHM-Natural History Museum of London, Department of Life Sciences, Cromwell Road, SW7 5BD London, UK; 16 University of Bergen, Department of Biological Sciences and K.G. Jebsen Centre for Deep Sea Research, PO Box 7803, 5020 Bergen, Norway; 17 Bangor University, School of Ocean Sciences, Menai Bridge, LL59 5AB Anglesey, UK; 18 ICM-CSIC-Institute of Marine Sciences, Passeig de la Barceloneta 37-49, 08003 Barcelona, Spain; 19 ICBM-Institute for Chemistry and Biology of the Marine Environment, University of Oldenburg, Schleusenstraße 1, 26382 Wilhelmshaven, Germany; 20 HIFMB-Helmholtz Institute for Functional Marine Biodiversity, University of Oldenburg, Ammerländer Heerstraße 231, 26129 Oldenburg, Germany; 21 CIIMAR-Interdisciplinary Centre of Marine and Environmental Research, University of Porto, Avenida General Norton de Matos, S/N, 4450-208 Matosinhos, Portugal; 22 University of Kiel, Christian-Albrechts-Platz 4, 24118 Kiel, Germany

**Keywords:** Environmental microbiology, Microbial ecology, Symbiosis

## Abstract

In the deep ocean symbioses between microbes and invertebrates are emerging as key drivers of ecosystem health and services. We present a large-scale analysis of microbial diversity in deep-sea sponges (Porifera) from scales of sponge individuals to ocean basins, covering 52 locations, 1077 host individuals translating into 169 sponge species (including understudied glass sponges), and 469 reference samples, collected anew during 21 ship-based expeditions. We demonstrate the impacts of the sponge microbial abundance status, geographic distance, sponge phylogeny, and the physical-biogeochemical environment as drivers of microbiome composition, in descending order of relevance. Our study further discloses that fundamental concepts of sponge microbiology apply robustly to sponges from the deep-sea across distances of >10,000 km. Deep-sea sponge microbiomes are less complex, yet more heterogeneous, than their shallow-water counterparts. Our analysis underscores the uniqueness of each deep-sea sponge ground based on which we provide critical knowledge for conservation of these vulnerable ecosystems.

## Abstract (web summary)

This study presents a large-scale analysis of microbial diversity in deep-sea sponges. They show that sponge microbial abundance status, geographic distance, sponge phylogeny and the physical-biogeochemical environment drive microbiome composition, in descending order of relevance. The uniqueness of each deep-sea sponge ground stresses the need for their strategic preservation.

## Introduction

Deep-sea sponge grounds (syn. aggregations, gardens) are sponge-dominated ecosystems that are found throughout the world´s oceans. These spatially extensive habitats enhance biodiversity^1^ and are nurseries and feeding grounds for commercially important fish species^2^. Deep-sea sponge grounds were identified as priority ecosystems^3,4^ that warrant protection against human interventions such as trawling or mining. As known hotspots of macrofaunal biodiversity, they modulate ecosystem dynamics and biogeochemical cycles, including nutrient cycling^5^ and the carbon pump^6^. Sponges are evolutionarily ancient animals, with sponge fossil evidence dating back 541–890 million years in time^7,8^. It is tempting to speculate that sponge symbioses are also ancient, but fossil evidence is lacking. Shallow-water sponges represent one of the most diverse and complex host-microbe associations in the marine environment, with more than 40 bacterial phyla, representing thousands of bacterial lineages in a single sponge individual^9^. While some sponges contain dense microbial consortia in their tissues (high microbial abundance (HMA) sponges), other species lack such dense communities (low microbial abundance (LMA) sponges)^10^. The microbial symbionts provide new functions to the sponge host, such as the expansion of the animal’s metabolic repertoire or defence against predators^11^. One current question is whether and to what extent the environmental context affects the stability of the host-microbe association. The ocean environment is rapidly changing and microbiome composition is directly related to sponge health and ecosystem function^11^, therefore reference baselines are urgently needed to monitor the integrity and resilience of sponge-dominated ecosystems.

While a significant body of information has been accrued on shallow-water sponges over the last two decades^9,10,12,13^, our understanding of deep-sea sponges and their associated microbes is still very limited. Existing studies on deep-sea sponge microbiomes have provided valuable insights into the microbial diversity and function for a handful of sponge species at a local scale^14–19^. Now the next frontier is to deduce general, global patterns in a synchronised way based on a larger variety of sponge species, and to establish a baseline in order to ensure a sustainable management of critical and threatened ecosystems. The deep sea is the largest biome on Earth, but its biodiversity and ecosystem dynamics are still underexplored. Less than 5% of the deep sea has been explored and less than 0.01% of the deep seafloor has been quantitatively sampled so far^20^. Our study aims to characterise microbial diversity in deep-sea sponges, and to determine the drivers that shape their community composition. Besides host- and environment-related factors, the effect of geographic distance between sites was explored. The resulting next-generation biodiversity assessment of deep-sea sponge microbiomes spans spatial scales from exploring individual sponge holobionts to an integrated ocean-wide assessment. To our knowledge, this is the largest analysis of host-associated microbial communities in the deep-sea. Further, our study is unique in the large variety of included environmental data. We have generated >50 metadata entries for each sample, spanning geographic, biogeochemical, and physical parameters. Our baseline dataset provides insights into the diversity, biogeography, and ecology of deep-sea sponge microbiomes at unprecedented spatial scales and further provides data-based directions for the conservation and management of the vulnerable sponge ground ecosystems.

## Results & discussion

### High diversity and taxonomic novelty in deep-sea sponge microbiomes

**Fig. 1:**
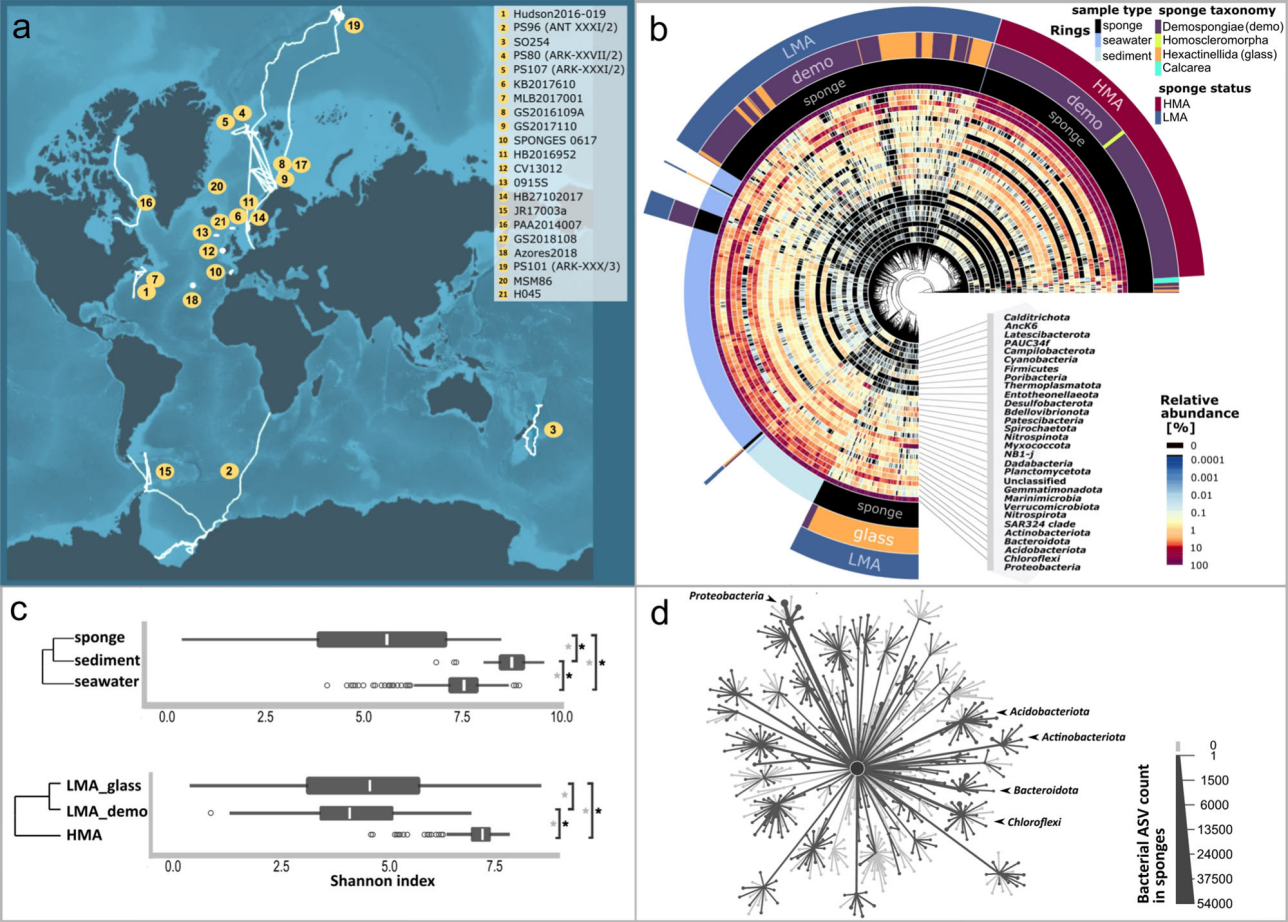
Microbial alpha- and beta-diversity in deep-sea sponges, seawater, and sediment; as well as expeditions overview. **a** Cruise tracks of 21 deep-sea expeditions conducted in the years of 2012–2019. Yellow labels mark cruise tracks, while exact sampling locations can be viewed here: 10.1594/PANGAEA.923033. **b** Similarity of microbial communities (beta-diversity) as illustrated by a circular clustering dendrogram based on weighted UniFrac distances. The outer coloured rings indicate sponge HMA-LMA status (Supplementary Figs. 1 and 2), as well as sponge taxonomy (Supplementary Data 1), and sample type. The relative abundances of the 30 most prominent microbial phyla are shown by a heatmap that is sorted with increasing average abundance from the inside to the outside. **c** Microbial richness (alpha-diversity), as expressed by Shannon indices of sample types (top) and sponge types (bottom). The following elements are shown in the box-plots: lower and upper box hinges = 25th and 75th percentiles; whiskers = 1.5× inter-quartile range extending from hinges; points = outliers; centre lines = median. The underlying numbers of independent biological samples (*n*) are: sponge = 931, seawater = 355, sediment = 108 for the top panel, and HMA = 359, LMA_demo = 311, LMA_glass = 243 for the bottom panel. Source data are provided as a Source Data file. Asterisks indicate statistical significance. Black asterisks show a significant pair-wise Dunn’s test (alpha-diversity) and grey asterisks a significant pair-wise PERMANOVA (beta-diversity). Both types of statistical tests were run two-sided, (*p*-values: Supplementary Tables 2, 3). The clustering dendrogram (weighted UniFrac distances) indicates overall similarity between sample types or between sponge types, respectively. **d** Microbial taxon richness of the entire deep-sea sponge dataset as illustrated by a heat tree. Each branch represents one of the 92 currently known bacterial phyla (including candidate phyla) which further splits into bacterial classes, as derived from the current SILVA database (version 138 SSU Ref NR 99). Those phyla and classes found in deep-sea sponges are coloured in dark grey (i.e.,^2^/_3_ of all known bacterial phyla). Bacterial taxa present in the SILVA database, but not in sponges are coloured in light grey. The sizes of nodes and lines are representative of the underlying bacterial ASV richness. The five most abundant bacterial phyla across all sponge samples are marked by arrows. Supplementary Fig. 3 shows a completely labelled version of the heat tree.

We tested the hypothesis that deep-water sponges associate with similar microbial communities as their shallow-water counterparts. Twenty-one deep-sea expeditions were undertaken with sampling campaigns at 52 sponge grounds primarily in the North Atlantic, but with representative samples from the Pacific, Arctic and Southern Oceans (Fig. 1a; Supplementary Table 1). This effort resulted in the collection of 1077 sponges (representing 169 sponge species), 355 seawater, and 114 sediment samples (with the latter two sample types serving as environmental “reference samples”). We herein describe the extent of diversity, specificity, and taxonomic novelty of microbes associated with these deep-sea sponges. The phylum Porifera consists of four taxonomic classes: Calcarea (calcareous sponges), Demospongiae (demosponges), Hexactinellida (glass sponges), and Homoscleromorpha. The hexactinellids, deep-sea sponges whose microbiomes remain understudied (but note^21,22^), constitute a significant fraction in our study (*n* = 243 sponges representing 56 species). We found that these glass sponges harbour a distinct microbiome, clustering apart from that of other sponges and also from environmental reference samples. Clustering of microbial communities by similarity revealed three main sponge groups, hereafter referred to as “sponge types” (Fig. 1b). These sponge types were further defined by combination of sponge taxonomy (Supplementary Data 1) and microbiome density. Microbiome density was determined based on light microscopy, transmission electron microscopy (Supplementary Fig. 1), and machine learning (following procedures of^10^, Supplementary Fig. 2). We termed these sponge types “HMA sponges”, “LMA demosponges (LMA_demo)”, and “LMA glass sponges (LMA_glass)”. The HMA-LMA dichotomy is well known from shallow waters where this status has also been linked to differences in pumping rates, carbon and nitrogen fluxes, and functional gene content between HMA and LMA sponges^10^. We now report here on a subdivision for LMA sponges into LMA_demo and LMA_glass sponges. In terms of alpha- and beta-diversity, we observed significant differences between the microbiomes of sponges compared to environmental reference samples (Fig. 1b, c and Supplementary Tables 2, 3). We also observed significant differences in microbial alpha- and beta-diversity between the three sponge types, where the LMA sponge types had an overall similar alpha-diversity. Overall, sponges harboured a lower microbial richness than environmental reference samples, and HMA sponges showed a significantly higher richness than LMA sponges. While those patterns are well known for HMA and LMA_demo sponges in shallow waters, we here expand the fundamental HMA-LMA dichotomy concept to deep ocean environments and show that LMA_glass sponges have their own characteristic microbiome.

The deep-sea dataset (including sponge and reference samples) contained 81 microbial phyla of which 71 occurred in sponges. Sixty-one of the sponge-associated microbial phyla were classified as members of the Bacteria, nine of the Archaea, and one of the Eukarya. Based on the SILVA database, we, therefore, recovered around ^2^/_3_ of all currently known bacterial phyla (including candidate phyla) in deep-sea sponges (Fig. 1d). Here we focus on amplicon sequence variants (ASVs, syn. features, which are the highest resolved grouping) for precise and reusable classification of microbial taxa. The 53,756 ASVs retrieved from sponges represented 201 bacterial classes, 379 orders, 463 families, and 747 genera. The five most abundant microbial phyla in sponges were Proteobacteria (on average = 47.6% relative abundance), Chloroflexi (15.8%), Acidobacteriota (8.4%), Actinobacteriota (4.7%), and Bacteroidota (3.5%), (Fig. 1d). Proteobacteria and Chloroflexi, as well as Anck6, Dadabacteria, Entotheonellaeota, Nitrospirota, PAUC34f, and Spirochaetota were significantly enriched in sponge compared to seawater and sediment samples (Supplementary Fig. 4). We detected 34% more microbial features and 30 more microbial phyla (with the newest SILVA reference database version 138 SSU Ref NR 99) than in a similar study on shallow-water sponge microbiomes^9^. A direct comparison between the two studies cannot be given without mentioning the caveats though, as both studies used different methods (e.g., different primer sets, sequencing methods, processing pipelines, sequencing clustering, and sampling depths). Besides the sheer microbial diversity, the number of unknown microbial taxa was remarkable (Supplementary Table 4). For example, 23,904 bacterial ASVs remained unclassified at the family-level, representing 44.5% of all sponge bacterial ASVs, and 50.4% of the average sponge community (averaged across all 931 sponges that remained in our dataset after all data filtering steps). Further, 2484 bacterial features were unclassified even at the phylum level. The high observed taxonomic novelty may be explained by microbial evolutionary processes within the sponge host, and by the understudied nature of the sampled biome, and the large size of the analysed deep ocean host-microbiome dataset.

### Individuality is the foundation of diversity

**Fig. 2:**
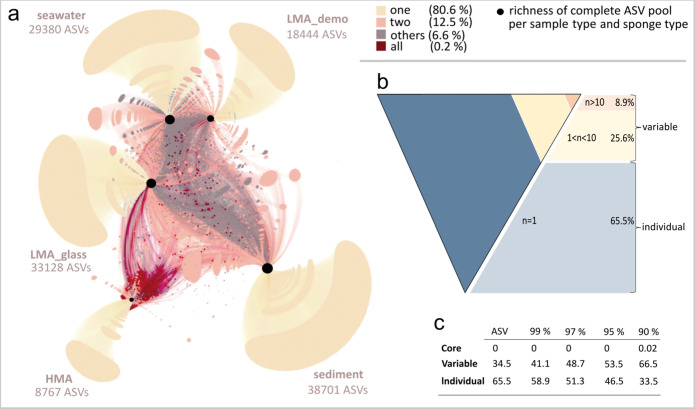
Microbial ASV distributions in deep-sea sponges and across sample types. **a** ASV distribution across sample types as illustrated by a bipartite network between sponge + sample types (HMA sponges, LMA_demo sponges, LMA_glass sponges, seawater, sediment) and microbial taxa. Total numbers of ASVs occurring in each sample type are given and reflected by size of black dots. Edge and node colours refer to the prevalence of ASVs in one (yellow-beige), two (orange-salmon), all (red) sample types. The category “others” (grey) denotes ASVs that do not fall in any category. **b** Tilted pyramid illustrates variable and individual fractions of the total ASV pool. “Individual” ASVs are defined as those occurring in one sample (blue), and “variable” ASVs as those occurring in 2–10 samples (yellow), or > 10 samples (orange). The variable community was split into two categories (ASVs occurring in 1 < *n* < 10 samples per type, or *n* > 10 samples per type) based on Supplementary Fig. 5 for the pyramid, while the two categories were merged in the table below (**c**). “Core” is defined as occurring in more than 70% of all sponge samples^87^ (i.e., 652 sponge samples). Values are given for different sequence clustering thresholds (amplicon sequence variants, 99% OTUs, 97% OTUs, 95% OTUs, and 90% OTUs, **c**).

Next we sought to explore how the ASVs are distributed among core, variable, or individual fractions of the microbiome. More than 80% of all ASVs were found in only one sample type (i.e., sponge, sediment, or seawater), whereas 0.2% of all ASVs were shared between all sample types (Fig. 2a). The fraction of ASVs shared between two sample types ranged from 1.4% (HMA sponges and sediment) to 16.2% (LMA_glass sponges and seawater), (Supplementary Table 5). Overall we observed a larger overlap between sponge and seawater microbial communities, than between sponge and sediment microbial communities. The pool of ASVs which occurred in less than ten samples of the same sponge type was large (>80–96% of all ASVs per sponge type; Supplementary Fig. 5). This finding is consistent with previous observations on, for example, surface marine planktonic microbiota^23^ and shallow-water sponges^9^. On average 65.5% of all ASVs occurred in only one sponge sample (Fig. 2b, these not being singletons, but occurring in multiple copies). We conclude that each deep-sea sponge individual carries its own set of microbes. Inter-individual differences between microbiomes have recently received notable attention in humans with respect to personalised medicine and nutrition strategies^24,25^. The observation of large variations in the microbial community composition of deep-sea sponges is further supported by a consistent lack of a core community across different sequence clustering thresholds (Fig. 2c). Only at a clustering threshold of 90%, two Operational Taxonomic Units (OTUs) fullfill the criterion of core community membership. These two OTUs were classified as characteristic deep-sea/seawater OTUs, corresponding to abundant and well characterised sponge symbiont clades: (i) Chloroflexi-Dehalococcoidia-SAR202_clade-hydrothermal_vent_metagenome and (ii) Actinobacteriota-Acidimicrobiia-Microtrichales-Microtrichaceae-Sva0996_marine_group. Mean relative abundances of ASVs were positively correlated with the number of samples in which the respective ASV occurred for HMA sponges and environmental reference samples (Supplementary Fig. 6), while there was no such relationship for LMA sponges. We suggest that core, variable, or individual community affiliation in deep-sea sponge microbiomes may be related to the strength of the host-microbe interaction^9^ or assembly mechanisms of microbial community members (deterministic vs. stochastic processes)^26^. The nestedness of a microbiome within an individual eco-evolutionary context together with a stochastic component and time may ultimately result in such highly individual assemblages.

### Sponge host drivers of microbial community composition

**Fig. 3:**
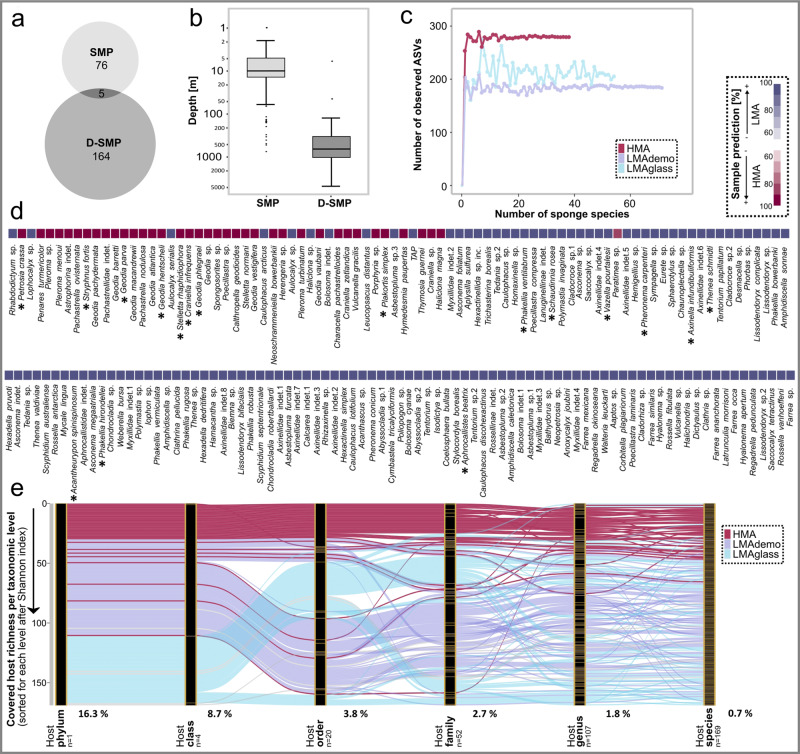
Microbial host-specificity, the sponge microbiome carrying capacity, and the HMA-LMA dichotomy in deep-sea sponges; as well as comparison to the Sponge Microbiome Project. **a**, **b** Overview over the Sponge Microbiome Project (SMP; reference dataset^9^) and the Deep-sea Sponge Microbiome Project (D-SMP; this study): **a** shows the differences in covered sponge species (*n* = 81 sponge species for the SMP, and 169 sponge species for the D-SMP), and b) shows the difference in sampling depth. The lower and upper hinges of the boxes correspond to the 25th and 75th percentiles and the whiskers represent the 1.5× inter-quartile range extending from the hinges. Points indicate outliers. The median is indicated in the boxplot as a solid line (at 10 m for the SMP, and 650 m for the D-SMP). *n* = 973 biologically independent samples for the SMP (804 sponges, 133 seawater samples, 36 sediment samples), and *n* = 1394 biologically independent samples for the D-SMP (931 sponges, 355 seawater samples, and 108 sediment samples). Source data for **a**, **b** is provided as a Source Data file. Figures **c**–**e** are based on the newly generated D-SMP dataset: **c** adapted rarefaction curves showing microbial richness plotted against the number of sponge species. **d** List of the 169 covered sponge species, sorted in descending order from left to right based on their respective microbial richness (Shannon index). Colouring indicates the predicted HMA-LMA status of each sponge species based on machine learning (Supplementary Fig. 2). Sponge species, which were inspected microscopically for their HMA-LMA status by transmission electron microscopy (*n* = 3 per species, Supplementary Fig. 1) are marked by an asterisk. All microscopically inspected sponge species were correctly classified by machine learning predictions (proof of concept; Supplementary Fig. 2). **e** Microbial community richness (Shannon index) at different host taxonomic levels and sorted anew by descending richness at each taxonomic level. Yellow lines mark boundaries of distinct taxonomic groups. Grey lines within the alluvial stand for ambiguous sponge status. Black percentages at the bottom of the plot indicate the median fraction of the host-specific ASV pool at the respective sponge taxonomic level. (Host-specific ASVs are defined as ASVs occurring only in one sample group of a given host taxonomic rank: e.g., in only one host species at the host species level, or in only one family at the host family level).

We queried to what extent the animal host shapes microbial community composition. Only two large-scale datasets on sponge-associated microbial communities are currently available: one published^9,27^, and the one presented here which includes twice as many sponge species. Figure 3a, b shows a comparison between the shallow-water Sponge Microbiome Project (SMP^9^), and the Deep-sea Sponge Microbiome Project (this study; D-SMP). While the average sampling depth of the SMP was 10 m, the average sampling depth of the D-SMP was 650 m. The covered sponge species largely did not overlap, which is consistent with shallow-water and deep-sea sponge species having different ecological ranges. Deep-sea sponge microbiomes had an overall lower complexity (number of microbial ASVs per sample; Supplementary Fig. 7) than previously recorded from shallow waters^9^. Specifically, we observed 22-1537 ASVs per host (average = 285) in deep-sea sponges, compared to the previously recorded 50-3820 OTUs (clustered at 97%, the expected ASV-level richness being even higher) in shallow-water sponges. However, one should take note of the previously mentioned caveats for a comparison between the two datasets due to differences in the applied methods. Although we observed some variability in deep-sea sponge-associated microbial richness, microbial alpha-diversity was remarkably constant within each sponge type across different world oceans, ocean zones, and geological settings (Supplementary Fig. 8). Adapted rarefaction curves were calculated to show microbial richness (number of observed ASVs) as a function of the number of observed sponge species (Fig. 3c). These adapted rarefaction curves displayed different saturation values, and we use the term “sponge microbiome carrying capacity” to describe the consistency of differences in microbial richness between the three sponge types. In ecology, the maximum microbial population size which can be sustained within a system is based on the available resources and typically referred to as carrying capacity (for example, see ref. 28). We postulate that the carrying capacity and consequently, microbial alpha-diversity in sponge-microbe associations is determined by resource limitation, resulting in constant patterns for each sponge type.

For this study, we have sampled 169 sponge species, which cover 107 sponge genera, 52 families, 20 orders, and 4 classes. The 169 sampled sponge species were classified as either HMA or LMA based on our machine learning analysis in combination with microscopic imaging (Fig. 3d and Supplementary Fig. 2). In total, 131 sponge species were classified as LMA sponges (56.8% LMA_demo, 43.2% LMA_glass) and 38 sponge species as HMA sponges. The HMA-LMA dichotomy was identified as a major driver of microbial community composition in deep-sea sponges similar to what has been reported for shallow-water sponges^10,27^. With regard to our deep-sea sponge collection, Chloroflexi, Acidobacteriota, Dadabacteria, Gemmatimonadota, Myxococcota, Entotheonellaeota, Spirochaetota, Poribacteria were the eight most enriched taxa in HMA over LMA sponges (Supplementary Fig. 4). In contrast, Proteobacteria, Bacteroidota, SAR324 clade, Planctomycetota, Verrucomicrobiota, Nitrospirota, Patescibacteria, and Marinimicrobia were the eight most enriched taxa in LMA over HMA sponges. While the overall HMA-LMA characteristic trends were validated in the majority of deep-sea sponge species, there were also a few noteworthy deviations from expected microbial alpha- and beta-diversity patterns (see Supplementary Note 1 for details). Microbial richness was consistently higher for the majority of HMA than for LMA sponges across all host taxonomic levels, whereas the variability in microbial richness was higher in LMA sponges (Fig. 3e).

**Fig. 4:**
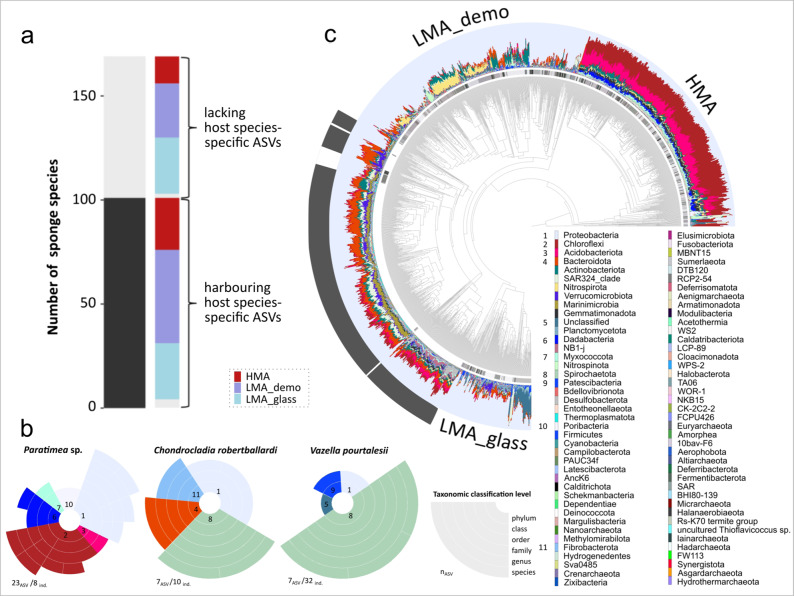
Sponge species-specificity and -exclusiveness of deep-sea sponge microbial communities. **a** Presence of sponge species-specific ASVs in the 169 sponge species analysed in this study. Coloured bars indicate distribution of presence (dark grey)/absence (light grey) of host species-specific ASVs across sponge types (red = HMA; blue = LMA_demo; light blue = LMA_glass). **b** Sunburst charts depicting sponge species-exclusive ASVs of one HMA sponge (*Paratimea* sp.), one LMA_demo sponge (*Chondrocladia robertballardi*), and the LMA_glass sponge *Vazella pourtalesii* (each with ≥8 sponge individuals per species). Rings indicate microbial taxonomic affiliation, from the inner (phylum) to the outer ring (species). When unassigned at a certain taxonomic level, colour was not added. Colour number code for microbial phyla is same in **b** and **c**, numbers clarify names of microbial phyla in **b**. Total numbers of species-exclusive ASVs are shown below each plot, together with the total number of sponge individuals per sponge species. **c** Relative abundances of the 81 microbial phyla (plus unclassified taxa) in all samples including seawater and sediment. The bar charts are sorted based on the similarity of microbial communities (beta-diversity; same order as in Fig. 1b). The grey shades of the ring, which is shown between the microbial clustering dendrogram and the bars, mark the 169 sponge species (different shades of grey denote different sponge species). Descriptors on the outer circle indicate the three sponge types (HMA, LMA_demo, LMA_glass), dark grey fill marks environmental reference samples (seawater, sediment). This plot provides a higher resolution of Fig. 1b: Relative abundances are shown for all detected 81 microbial phyla, as well as for taxa that are unclassified at phylum level. Information about sponge species identity is also included.

Sponge taxonomy was identified as another major driver of microbial community composition, which is in line with previous reports from shallow-water sponges^27,29^. In deep-sea sponges, the effect on alpha- and beta-diversity was particularly evident on the host phylum, class, and order level, while at lower host taxonomic levels patterns became less clear (Fig. 3e and Supplementary Fig. 9). This is probably a consequence of increasing sample heterogeneiety outweighing the host signal at lower taxonomic ranks. In order to analyse microbial specificity patterns on lower host taxonomic ranks, we determined “host-specific ASVs”, defined as those occurring only in one sample group of a given host taxonomic rank (Fig. 3e; e.g., occurring in one host species/genus/family/order/class only), and lacking in the environmental reference samples. 101 out of 169 sponge species harboured such host-specific ASVs (Fig. 4a). When querying for ASVs that were both occurring only in one sample group of a given host taxonomic rank and occurring in >90% of all samples per group (hereafter termed “exclusive ASVs”), 66 sponge species were identified (Supplementary Data 2). The microbial composition of the exclusive ASV pool was assessed in more detail for 3 selected sponge species with characteristic lifestyles/morphotypes, (the demosponge *Paratimea* sp. having an unusually rigid outer coating, the carnivorous sponge *Chondrocladia robertballardi*, and the glass sponge *Vazella pourtalesii* occurring monospecifically on sponge grounds), (Fig. 4b). Here, the exclusive ASV pool was composed of phyla that were also numerically dominant in the respective host species (e.g., Poribacteria in *Paratimea* sp., Bacteroidota for *Chondrocladia robertballardi*, and Patescibacteria in *Vazella pourtalesii*), indicating that these are likely functionally relevant bacteria for the sponge host. Out of the 169 sponge species, 68 lacked species-specific ASVs, of which 53 were LMA sponges (Fig. 4a). Despite the lack of species-specific ASVs, these sponge species overall still displayed species-specific microbiome compositions in terms of relative abundances (Fig. 4c), highlighting the role of both HMA and LMA sponges as highly specialised microbial reservoirs. We thus observed microbial specialisation on two levels, presence and enrichment of microbial specialists in sponges with unique lifestyles, and of microbial generalists that probably fulfil more generic functions in the corresponding sponge types (HMA, LMA demosponges, LMA glass sponges). One prominent example are the Chloroflexi, which are characteristic indicator phyla of HMA sponges (Fig. 4c and Supplementary Fig. 4) and also highly specialised symbionts of *Paratimea* sp. (Fig. 4b). Chloroflexi are well-described sponge symbionts that engage in degradation of dissolved labile and recalcitrant organic matter^30,31^. Consistent with the increasing depth profile of pelagic Chloroflexi, we find higher relative abundances within deep-sea sponges over those in shallow waters^31^, given the previously mentioned caveats of comparisons between disparate datasets.

### Distance–decay relationships

**Fig. 5:**
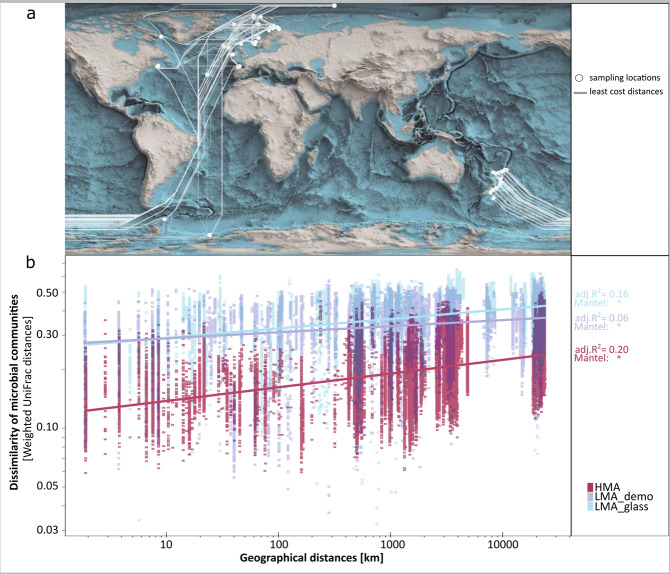
Geographic structure of deep-sea sponge microbiomes: Distance–decay relationships. **a** Least cost distances (below 200 m water depth) are shown between all 52 sampling locations. **b** Dissimilarity of microbial communities plotted against geographic distances for all analysed HMA, LMA_demo, LMA_glass sponges on a log–log scale. Values next to the plot refer to statistical outputs of regressions (adj. *R*²), and Mantel tests between dissimilarity and distance matrices (two-sided). Asterisks mark significant outputs of Mantel tests, the exact *p*-values are as follows: HMA = 0.001; LMA_demo = 0.017, LMA_glass = 0.001.

Significant distance–decay relationships have previously been reported for seawater and sediment microbial communities. These have been attributed to a limited capacity for long-distance dispersal of microbes in the deep-sea^32,33^. Taking advantage of the global collection effort spanning distance ranges of 10 to >10,000 km, our study analyses distance–decay relationships at an unprecedented scale for sponges. We observed that deep-sea sponge-associated microbial community dissimilarity increased weakly, but significantly with increasing geographic distance for all three sponge types (Fig. 5). We propose that the observed distance–decay relationships in sponges are linked to isolation by distance on at least two hierarchical levels: (i) limited long-distance dispersal capacity of sponge larvae, impacting sponge species distributions and thus geographic patterns of vertically transmitted microbes, and (ii) limited long-distance dispersal capacity of environmental reference microbiomes, imprinting biogeographic patterns on the horizontally-acquired fraction of the sponge microbiome. Our results thus imply that sponge microbiomes exhibit a subtle biogeography which is likely shaped by a limitation of contemporary long-distance larval dispersal processes in addition to local selection processes. Indeed, location turned out to be the second most deterministic factor for explaining microbial variability in deep-sea sponges. Results of overall variation partitioning modelling, which was conducted in order to parse variation across all factors, revealed the following main drivers of microbial variability in deep-sea sponges in descending order: the sponge status (HMA-LMA; 3.9% of constrained variation), location (2.0%), host phylogeny (1.3%), and environmental cluster (0.7%).

### Environmental drivers of sponge microbial community composition

**Fig. 6:**
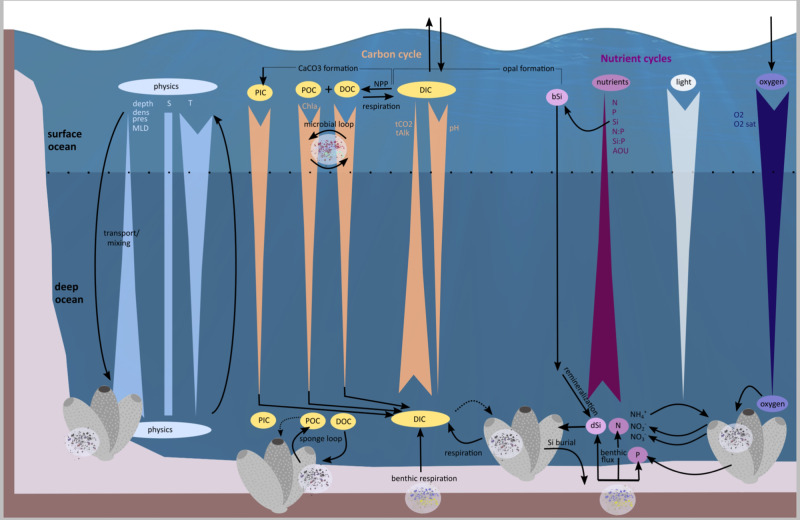
Factors and processes in the ocean ecosystem. Schematic representation of physical and biogeochemical gradients over depth and sponge engagement in major biogeochemical processes^6,34^. This framework serves as the basis for our multifactorial analysis to determine environmental drivers of sponge-associated microbial community composition. Physical parameters include salinity (S) and temperature (T) among others. Biogeochemical parameters include those involved in the sponge loop (dissolved organic carbon (DOC) and particulate organic carbon (POC)), those representing the major nutrient cycles (e.g., nitrogen (N), silicon (Si), and phosphorus (P)), as well as oxygen concentrations (O_2_). See Supplementary Table 6 for details and abbreviations.

**Fig. 7:**
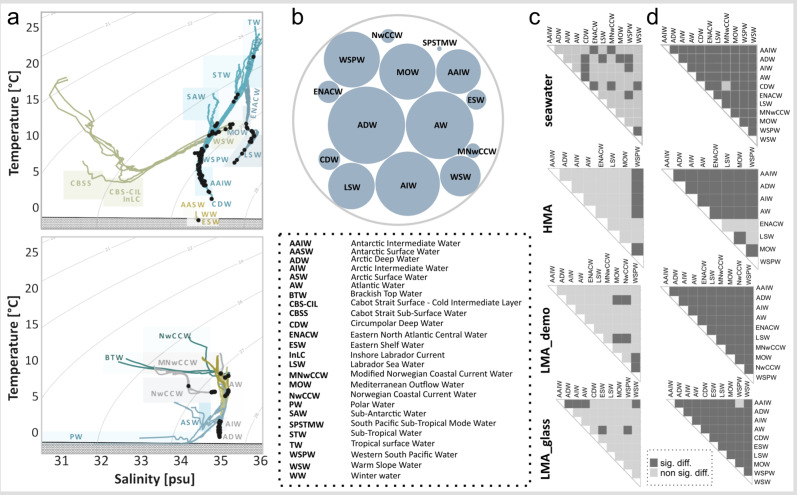
Water masses and their relation with microbial alpha- and beta-diversity in deep-sea sponges. **a** Temperature-salinity (T-S) profiles derived from 66 conductivity-temperature-depth (CTD) casts in different ocean regions. Presented records start 20 m below the ocean surface and reach down to 5 m above the ocean floor. Boxes indicate characteristic T-S—ranges of prevailing water masses, which were classified manually based on literature^35,37–48^. The full names of all water mass abbreviations are listed in the dashed box. Black points indicate sampling depths of sponges (indirectly, by marking the measured in situ temperature and salinity values for the sampled sponges with dots on the respective T-S profile). The individual profiles were pseudo-coloured and allocated into two separate panels to enhance readability. Dashed lines represent density lines (sigma theta, σθ = [kg/m³]) and the lines which are filled with circles at the bottom of each graph, represent freezing point lines. **b** Number of samples derived from each water mass, encoded by circle size. The bigger the circle, the higher the number of samples. **c** Visual representation of statistical testing results (Dunn’s tests) to assess variations in microbial alpha-diversity (Shannon index) between water masses. The details of statistical testing are listed in Supplementary Data 3. **d** Statistical testing (PERMANOVAs based on weighted UniFrac distances) to assess differences in the microbial community composition between water masses evaluated at a significance level of α = 0.05. Further details of statistical testing are compiled in Supplementary Data 4.

In times of rapid environmental change, knowledge about how biological communities are linked to surrounding environmental conditions is key to assess their rarity and resilience. Sponges play a major role in biogeochemical cycles^6,34^ (Fig. 6), and their host community compositions and densities are impacted by the prevailing physical and biogeochemical conditions^35,36^. Here, we explored the variations of sponge microbial communities between natural environmental boundaries. In total, we determined 25 water masses manually from 66 generated CTD profiles (literature^35,37–48^ served as reference for water mass identification). Sponges and environmental references were sampled from 14 of these water masses (Fig. 7a and Supplementary Table 7), with the largest fraction originating from Arctic Deep Water (ADW; 20.9% of all samples); Atlantic Water (AW; 16.0%), and Arctic Intermediate Water (AIW; 14.5%), (Fig. 7b). Microbial alpha-diversity remained mainly constant across water masses for all sponge types and seawater (Fig. 7c and Supplementary Data 3), while significant differences were observed in the microbial community composition between water masses in almost all cases (Fig. 7d and Supplementary Data 4).

**Fig. 8:**
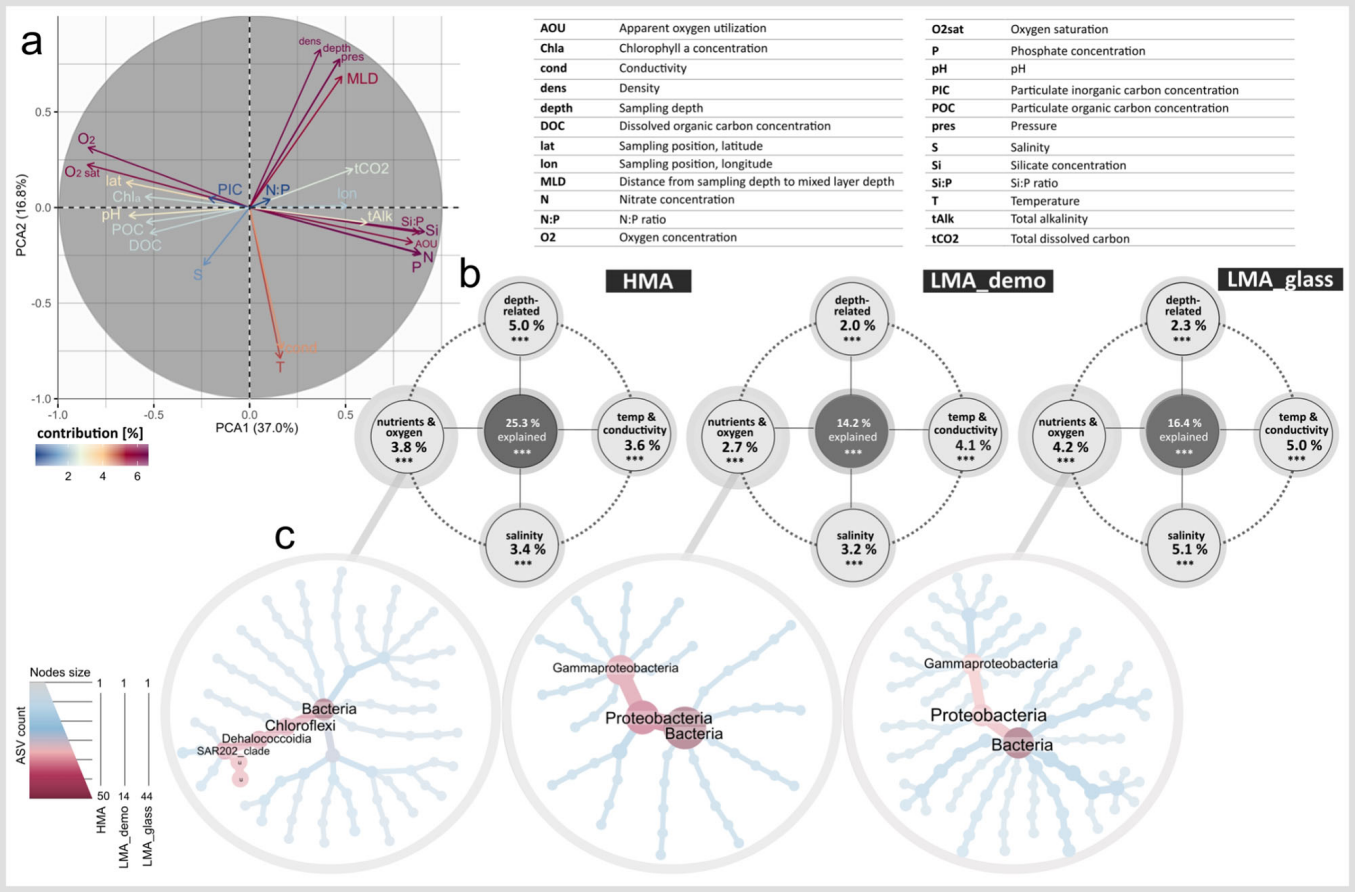
Environmental drivers of microbial diversity in deep-sea sponges. **a** Principal component analysis (PCA) of 24 environmental parameters. Colouring is ramped according to the contribution of each parameter. Full names for abbreviations of environmental parameters are given in the upper part of the plot. **b** Final variation partition models for HMA sponges, LMA_demo sponges, and LMA_glass sponges. Asterisks indicate the significance of models as assessed by permutations. Percentages indicate the fraction of microbial variability that is explained by the four parameter groups individually, and together (center of each sub-plot). Note that only those microbial taxa which occurred in more than 10 samples of each sponge type were considered for this analysis. **c** Heat trees of microbial community compositions occurring in the nutrient/oxygen modules of HMA sponges, LMA_demo sponges, and LMA_glass sponges. Corresponding modules were derived from weighted gene correlation networks. Only those taxa with a modularity >0.8 are shown, as these taxa show strongest connections to other taxa in the network as well as strongest correlations to nutrient and oxygen concentrations. Colours and node sizes in the heat trees indicate abundance of respective microbial taxa. Unclassified taxa are abbreviated with “u”, and only the most abundant taxa are labelled.

In order to evaluate the variability of deep-sea sponge-associated microbiomes in relation to environmental conditions, we compiled 24 environmental parameters (Fig. 8a, Supplementary Table 6, and Supplementary Fig. 10). Co-varying parameters were grouped into environmental driver categories during data analysis (see Method section for details). Depth-related parameters, temperature-related parameters, salinity, as well as nutrient (N, P, Si), and oxygen concentrations were identified as the main environmental drivers of microbial variability in deep-sea sponges (Fig. 8b). Correlations between microbial community compositions (weighted UniFrac distances) and each single environmental parameter behind these four categories (Euclidean distances) were statistically significant (Supplementary Data 5). While physical parameters (temperature, salinity, and depth) have previously been identified as relevant drivers of host-associated and free-living microbial communities^12,49–51^, we add here an extended suite of biogeochemical parameters that together with water mass properties provide a comprehensive view on the abiotic context across multiple scales up to an ocean-spanning scale. We observed a modular structure of the microbial community composition, in the sense that the overall microbial community is divided into multiple sub-groups, in which members have particularly high putative interactions among each other. A modular structure of the microbial community has previously been proposed to enhance robustness against perturbations in shallow-water sponges^12^. Those microbial taxa which responded most strongly to environmental gradients were generally also those taxa which were the most dominant members of the microbial community (Fig. 8c). This was the case for all main environmental drivers at high taxonomic levels (i.e., microbial phylum, class) (Supplementary Fig. 11). We conclude that modularity of deep-sea sponge microbiomes is directly linked to high-level taxonomic stability of the microbial community within sponge types. This implies that variations in the microbial community composition upon changing environmental conditions may not be detected on high taxonomic ranks. However, we observed notable differences in the modular taxonomic composition between both the sponge types, and the main environmental driver sets, at lower taxonomic ranks (i.e., below the microbial class level; consider light blue lines in Fig. 8c and also Supplementary Fig. 11). Different microbial strains are known to display functional redundancy, but may also diversify with selective factors, which can lead to a decoupling between taxonomic and functional complexity^9,52,53^. Generally, broad functions (such as carbon catabolism) are considered to be more functionally redundant than narrow functions (such as specific compound degradation), resulting in an increased buffering capacity against taxonomic shifts induced by biotic or abiotic disturbances (ref. 54 and references therein).

The four main identified environmental driving forces (temperature, salinity, depth, and nutrients/oxygen) explained 25.3% of the variability in HMA sponges, 14.2% in LMA demosponges, and 16.4% in LMA glass sponges. We observed a higher percentage of explained variation in HMA sponges despite a higher overlap of microbial features between LMA sponges and seawater in this and previous studies (Supplementary Table 5 and ref. 55). One explanation may be a higher uniformity of HMA sponges (more microbial phyla occurring across multiple samples) and the fraction of specific ASVs being higher in HMA (28.2%) over LMA sponges (10.7% LMA_demo; 8.0% LMA_glass). Although the degree of intimacy of the host-microbe interaction varied between sponge types, a considerable fraction of the microbial community was shaped by environmental factors in all sponge types. The two environmental drivers temperature and oxygen have recently received special attention with respect to future ocean conditions^56,57^. It has been estimated that ~80% of the predicted oxygen loss will occur in the deep-sea, leading to increased respiratory oxygen demand at some geographic locations of the deep ocean^56^. In addition, particularly in areas of deep-water formation such as the North Atlantic Ocean, the effects of sea surface warming may reach down to the seafloor and impact the vulnerable deep-sea sponge ground ecosystems^36^.

### Conservation of deep-sea sponge ground ecosystems

**Fig. 9:**
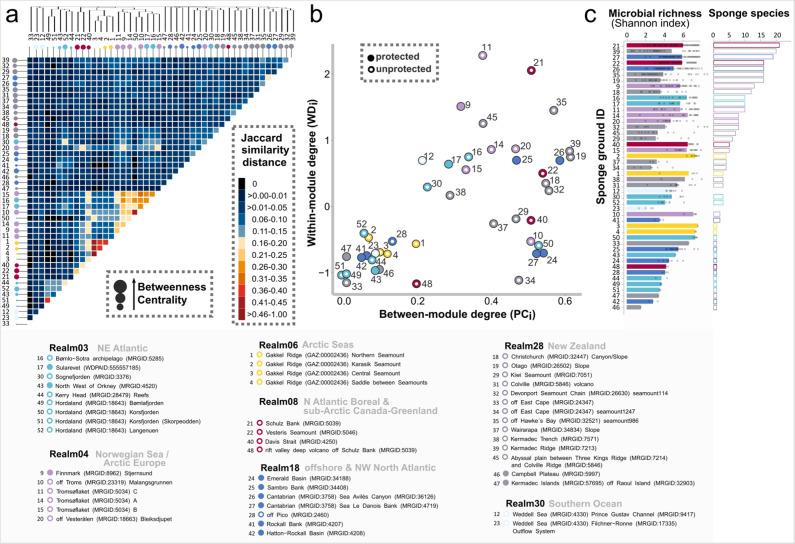
Variations in sponge-associated microbial diversity between deep-sea locations. **a** Dissimilarity of the microbial community composition between sampling locations. The heatmap encodes a similarity network between the analysed sponge ground locations based on Jaccard distances (presence-absence of microbial taxa). Locations are sorted after Jaccard similarity (by complete linkage clustering) where numbers encode for location names (compare text below plot). Coloured dots indicate the realm (based on ref. 84) of the respective sponge ground. Dot sizes encode for node betweenness centrality, i.e., the centrality of the respective location in the constructed similarity network. **b** Connectivity of microbial community compositions between sponge grounds. Within- and between-module degrees of different sponge grounds as derived from a bipartite network between microbial taxa and sponge grounds (location). Full circles indicate protected sponge grounds, while rings encode for unprotected locations. Source data are provided as a Source Data file. **c** Microbial richness (Shannon index) of sponge-associated microbiomes at each location. Locations are sorted in descending order following the observed sponge species richness found at each site. Source data are provided as a Source Data file.

Conservation of biodiversity in the open ocean is a major current challenge to human-kind^58^ and it is considered a pressing need to secure ocean services (such as food provision, natural products, and climate regulation) for the generations to come. The microbial baselines established here for deep-sea sponge ground ecosystems are highly relevant for the documentation of their integrity and resilience in the long run^59^. In order to assess microbial similarity between sponge grounds, we established a similarity network between locations (Fig. 9a), and a bipartite network between locations and microbial feature occurences (Fig. 9b). We observed an overall low similarity and connectivity of the microbial community composition between locations. Individual sponge grounds were different in microbial beta-diversity and in total microbial alpha-diversity per location (Fig. 9c and Supplementary Fig. 12). The observed differences in alpha-diversity between sponge grounds are most likely linked to differences in the prevailing sponge community compositions, as statistical analyses revealed that alpha-diversity was constant between sites in almost all cases when considering each sponge type separately (Supplementary Fig. 12). Harbouring many specialist microbial taxa, each sponge species (or even sponge individual) represents a unique microbial ecosystem, which should not only be considered on the macro-, but also on the micro-level. This nestedness of microbial communities inside a host with an individual eco-evolutionary history prevents the formulation of a simple relationship between biogeographic scale and microbial similarity of sponge grounds, and highlights the need to include sponge diversity in conservation assessments. When doing so and considering each sponge type separately, the microbial community compositions were significantly different between realms (Supplementary Fig. 13), showing that biogeographic imprints are likely driven by isolation by distance and environmental selection. Overall, sponge microbiomes occurring in the same ecological realm were more similar to each other than to more distant grounds (Fig. 9a), although some proximate locations within realms remained highly dissimilar (e.g., 24 and 25; 26 and 27). These aspects imply a need for basin-scale protected area networks within ecological realms. In order to define priority areas for conservation of deep-sea sponges and their associated microbiomes at such large spatial scales, the constituent sponge grounds can be chosen by considering network connectivity (within-module degree and between-module degree; Fig. 9b), and at smaller scales, microbial richness at the site can be used to prioritise those selections (Fig. 9c). Establishment of networks of protected areas across these spatial scales will require concerted politics and decision-making between nations whose jurisdictions fall within these large ocean realms, but also the engagement of the global community for areas that fall beyond national jurisdictions.

We urge that the entire sponge holobiont (the animal and the associated microbiome) should be considered when designing and implementing conservation strategies for sponge ground ecosystems. This ideally entails protection of individual sponge species with a particularly diverse microbial community, highly specific key microbial taxa, and those with a high susceptibility for altered environmental conditions (e.g., via mining activities) among others. Unfortunately, the total space needed for protected areas is in stark contrast to the low number of currently protected sites (Fig. 9b). We propose that a much larger number and/or size of sponge ground conservation areas will be required to provide critical ecological services and to ensure resilience of deep-sea ecosystems in the long run. The high diversity of sponge holobionts detected in this study argues for a larger proportion than the current political goal for protecting 30% of the ocean by 2030 to safeguard biodiversity and build ocean resistance to environmental change.

### Concluding summary of the presented Deep-sea Sponge Microbiome Project results

Identifying the extent of unknown biodiversity in remote areas such as the deep ocean is one of the current frontiers in biology, but is hampered by a lack of synchronised large-scale sampling efforts in these regions. Based on our global standardised collection effort, we report sponges to be highly diverse, taxonomically novel, and specialised microbial reservoirs in the deep-sea. The enigmatic and understudied glass sponges were shown to have their own distinct LMA microbiome profile. Based on the novel assignment of 169 deep-sea sponge species into either HMA or LMA categories, we conclude that the HMA-LMA concept, a long-standing paradigm in sponge microbiology, applies to the deep ocean, despite a minimal overlap in analysed sponge species between shallow and deep waters, and despite a low contemporary connectivity between individual sponge grounds.

When comparing microbial diversity of deep-sea sponges versus shallow (which cannot be done precisely given the previously mentioned methodological considerations), we found that similar microbial indicator phyla were present. Many novel lineages were discovered, of which some were even unclassified on phylum level. Chloroflexi were generally present in higher relative abundances than in shallow-water sponges. We found that the microbiomes of deep-sea sponges were less complex (in terms of alpha-diversity) and more heterogeneous (in terms of beta-diversity). The nested sampling design revealed a similarly modular microbiome structure as has been observed in shallow-water sponges. While the overall structure of deep-sea sponge microbiomes resembled that of shallow-water sponges, the high variability in beta-diversity yielded still individually unique microbial compositions.

The sponge microbial abundance status and sponge taxonomy were identified as main host drivers of microbial community composition in deep-sea sponges. By introducing the concept of exclusive ASVs, we identified highly intimate sponge-microbe associations, particularly in sponges with characteristic lifestyles and morphotypes. In terms of environmental factors, temperature, salinity, depth, and nutrients/oxygen were identified as basin-scale drivers of sponge microbiome composition, together explaining up to 25.3% of microbiome variations in sponges. We further revealed that the surrounding water masses and geographic distance have an imprint on sponge microbiome composition on a global scale. A ranking of the main driving factors revealed the sponge status (HMA-LMA) to be the primary factor driving microbial variability in deep-sea sponges, followed by location, host phylogeny, and environmental cluster. In summary, our results highlight the need to consider the ecological context of host-microbe associations in order to comprehensively resolve patterns and drivers of microbial composition and structure. This cumulative knowledge base serves as a guideline for science-based management strategies for the conservation of vulnerable deep-sea sponge ground ecosystems.

## Methods

Strict standard operating procedures (SOPs) were established to reduce technical variation to a minimum. The wet-lab standard operating procedure was archived at protocols.io^60^. The computational script of our core bioinformatic pipeline (incl. visualisations of technical validations) was deposited on github: https://kathrinbusch.github.io/16S-AmpliconCorePipeline/^61^. The research presented here complies with all relevant rules and regulations regarding the access to samples and their import/export.

### Ocean going expeditions, *16S* rRNA gene amplicon sequencing, and statistical analyses

The presented data were obtained from 21 ship-based expeditions in the years 2012–2019 (Supplementary Table 1). Sponge samples were collected from depths between 6 and 4833 m depth. The median sampling depth across all samples was 650 m. Most sponges in this dataset were sampled at depths > 200 m, with the few individuals sampled from < 200 m also included and referred to as “deep-sea sponges” as they spanned characteristic deep-sea sponge species. By contrast, sponges included in the previous SMP (ref. 9) were mainly sampled from depths < 200 m and are referred to as “shallow-water sponges”. Fifty-two sponge ground locations predominantly in the North Atlantic, the Arctic Ocean, Southern Ocean, and the South-West Pacific were probed during 271 sampling events. After the filtering steps, 46 sponge ground locations were retained in the analyses. Our filtering steps included: (i) a removal of sponges with an ambiguous host taxonomic identification, (ii) a removal of contaminated samples (based on unrobust microbial fingerprints), and (iii) a removal of samples with less than 5000 reads (for more details on the bioinformatic filtering steps and quality criteria see https://kathrinbusch.github.io/16S-AmpliconCorePipeline/). Altogether, 1077 sponge individuals, 355 seawater samples and 114 sediment samples were collected and processed in a standardised way. Following removal of samples that did not pass our quality criteria, 931 sponges, 355 seawater samples, and 108 sediment samples (1394 samples in total) were included for subsequent analyses (Supplementary Data 10).

For *16S* amplicon sequencing, DNA was extracted in a standardised way at the GEOMAR laboratory by using the DNeasy PowerSoil Kit (Qiagen; see Supplementary Data 11 for dates of DNA extraction). The V3-V4 variable region of the *16S* rRNA gene was amplified using the primer pair 341F-806R^62,63^ and sequenced on a MiSeq platform (MiSeqFGx, Illumina, San Diego, CA, United States) at the Competence Centre for Genomic Analysis (CCGA) Kiel. The respective primer sequences have been uploaded to protocols.io^60^. Raw reads were archived in NCBI within an Umbrella BioProject: PRJNA664762. Reads were processed within the QIIME2 environment^64^ (version 2019.10). Amplicon sequence variants (ASVs) were generated using the DADA2 algorithm^65^. Removal of singletons and chimeric sequences was performed and phylogenetic trees were calculated (FastTree2 plugin). For taxonomic classification of representative ASVs, a primer-specific trained Naïve Bayes taxonomic classifier, based on the SILVA 138 99% OTUs *16S* database^66^, was used. Mitochondria, chloroplasts and sequences unassigned at the domain-level were removed during taxonomic filtering steps. A sampling depth of 5000 was applied to standardise the number of reads across samples, at which point the rarefaction curves were saturated. A total of 27,815,393 reads (equal to 77% of all input reads) remained. Visualisations were done using R^67^ (versions 3.0.2, 3.5.1, and 3.6.2), Inkscape (version 0.92.4), python (version 3.7.3), Anvi’o^68^ (version 6.2), QGIS (version 3.4.4), Blender (version 2.92.0), and gephi (version 0.9.2). Supplementary Methods 1 contains a detailed overview about all statistical analyses conducted and Supplementary Methods 2 provides additional explanatory text. In brief, we worked with four different alpha-diversity metrics (Shannon index, Faith’s phylogenetic diversity, Pielou’s evenness, and number of ASVs). Due to an overall consistency between these metrics, we focused only on the Shannon index for statistical testing (Dunn’s tests). In terms of beta-diversity, we focused on weighted UniFrac distances for statistical analyses (i.e., pair-wise PERMANOVAs, sample clustering dendrograms, and Mantel tests). For the establishment of a similarity network between sponge grounds we used Jaccard (dis-)similarities. ASV abundance tables were standardised by either using relative abundances or presence–absences. Presence–absence data was used for bipartite networks between ASVs and locations, or between ASVs and sample types. Relative abundance tables were used for redundancy analyses, in variation partitioning models, and for weighted gene correlation networks. Relative abundance tables combined with taxonomic annotation (on the microbial phylum level) were used in Linear Discriminative Analyses. In addition, relative abundance tables on the microbial phylum- and class-level were used for the applied machine learning approach, using the Random Forests algorithm^10^. Core, variable, and individual microbial community members were determined: “Individual” ASVs occur in only one sample, “variable” ASVs occur in 2- 651 samples, “core” ASVs occur in > 652 sponge samples (i.e., in more than 70% of all sponge samples). No relative abundance thresholds were applied in conjunction with occurrence in number of samples (but singletons had been removed earlier as described above). Representative sequences were clustered on the ASV-level, 99% OTU-level, 97% OTU-level, 95% OTU-level, and 90% OTU-level in order to evaluate core, variable and individual memberships across different clustering thresholds. In order to determine drivers of microbial community composition, microbial ASVs were filtered based on the following criteria for several analyses: (i) to evaluate the specificity of microbial taxa, all features belonging to the “individual” fraction were not included, (ii) to analyse environmental drivers of the microbial community composition, only those microbial taxa which occured in more than 10 samples of each sponge type (HMA, LMA demosponges, LMA glass sponges) were considered. “Specific” ASVs were determined on different host taxonomic levels, and refer to those ASVs which occur only in one group at the respective host taxonomic level (e.g., in one species at the host-species level, or one family at the host-family level). The term “exclusive” ASVs was introduced, in order to describe specificity on different host taxonomic levels. Exclusive ASVs refer to ASVs that were both occurring only in one sample group of a given host taxonomic rank and occurring in >90% of all samples per group. Adapted rarefaction curves were created, showing microbial richness (number of observed ASVs) as a function of the number of observed sponge species. For these curves a random, steadily increasing (*n* + 1) set of sponge species was chosen until the maximum number of sampled species was reached. Note that for every step (*n* + 1), the samples were chosen based on the complete sponge species set, irrespective of the species covered in the previous step. We hence refer to the resulting curves, showing microbial richness plotted against the number of sponge species, as “adapted rarefaction curves”. With the help of a redundancy analysis (RDA), we determined the main environmental drivers of microbial community composition in deep-sea sponges. To avoid collinearity among environmental factors, explanatory variables with the highest variance inflation factor were removed sequentially during the RDA analysis procedure. Geographic distances between samples and between sampling locations were calculated as the shortest path by sea below 200 m water depth with the help of the R package “marmap” (ref. 69; version 1.0.5), only allowing connecting routes through water. Distance–decay relationships were examined based on geographic distances and microbial dissimilarities (weighted UniFrac distances), both log-transformed. Besides regressions, Mantel tests were conducted to assess these relationships statistically. In order to rank the different driving factors of the variability in deep-sea sponge microbiomes, overall variation partitioning models were set-up and run including all factors, i.e., sponge status (HMA-LMA), location, host phylogeny, and environmental parameters. For more details on this analysis, see Supplementary Methods 2. A significance level of α = 0.05 was applied to all statistical analyses in this study.

### Sponge taxonomy

Preliminary taxonomic assignments were made on board ship by leading sponge taxonomy experts, often in combination with in situ photographs, validated at a later stage by leading taxonomic experts and standardised with help of the World Register of Marine Species^70^ (WoRMS) by using Aphia IDs. Aphia IDs were provided at higher taxonomic levels when species-level identities were not possible. A combination of barcoding (*18S*, *COI* sequencing) of representative individuals was performed along with morphological analyses of sponge spicules. All four sponge classes were sampled, covering 20 sponge orders, 52 sponge families, 107 sponge genera, and 169 sponge species. Most of the sponge species studied here belonged to the two classes Demospongiae (110 sponge species) and Hexactinellida (56 sponge species), while only few sponge species were classified as Calcarea (2 sponge species) or Homoscleromorpha (1 sponge species).

### Tissue imaging

Ultra-thin (70 nm) and semi-thin (0.5 µm) tissue sections were generated for 17 sponge species (*n* = 3 each) in order to visually assess the HMA vs LMA status^10,71^. Tissue samples were fixed onboard ship in 2.5% glutaraldehyde in 0.1 M natriumcacodylate buffer (pH 7.4; Science Services GmbH). Back in the home laboratory, samples were rinsed with buffer 3× at 4 °C, post-fixed for 2 h in 2% osmiumtetroxide (Carl Roth), and washed with buffer (3 × 15 min at 4 °C). Samples were dehydrated with an ascending ethanol series (2 × 15 min 30% EtOH, 1 × 15 min 50% EtOH, storage at 70% EtOH), (ROTIPURAN® Carl Roth). After overnight storage at 4 °C, desilicification was performed with 4% suprapure hydrofluoric acid (Merck) for 5 h. The samples were washed thoroughly (8 × 15 min in 70% EtOH) with overnight storages at 4 °C between washing steps. Samples were further dehydrated (1 × 15 min 90% EtOH, then 2 × 15 min 100% EtOH) and gradually infiltrated with LR-White resin (AgarScientific) at room temperature (1 × 1 h 2:1 Ethanol:LR-White; 1 × 1 h 1:1 Ethanol:LR-White; 1 × 1 h 1:2 Ethanol:LR-White; 2 × 2 h pure LR-White). Following overnight incubation in pure LR-White at 4 °C, the samples were transferred into fresh resin within embedding capsules, that were polymerised at 57 °C for 2 days. After manual trimming, sections were cut (with at least three technical replicates) with an ultramicrotome (Reichert-Jung ULTRACUT E, equipped with a diamond knife (DIATOME, Switzerland)). Ultra-thin sections were cut at 70 nm thickness, mounted onto pioloform coated copper grids (75 mesh; Plano), and contrasted with uranyl acetate (Science Services; 20 min incubation with subsequent washing steps) and Reynold’s lead citrate (Carl Roth; 3 min incubation with subsequent washing steps). The ultra-thin sections were inspected on a Tecnai G2 Spirit BioTwin transmission electron microscope (FEI Company) using an acceleration voltage of 80 kV. Semi-thin section were cut at 0.5 µm thickness, stained with Richardson solution (Carl Roth), and visualised with an Axio Observer.Z1 microscope (Zeiss, Germany).

### Contextual data

Sixty-six full water conductivity-temperature-depth (CTD) profiles were conducted in different ocean regions and archived in the Pangaea database^72^. Profiles were trimmed to a starting depth of 20 m below the ocean surface and reached down to ~ 5 m above the ocean floor. Based on the resulting temperature-salinity profiles, prevailing water masses were classified manually with the help of literature^35,37–48^. In total, 24 environmental parameters were gathered in this study. Supplementary Table 6 provides a detailed overview on which parameters were included and by which method they were retrieved. Those parameters that were not measured in situ, but derived from climatologies, originate from three sources: (i) the World Ocean Atlas (WOA; version WOA18; refs. 73–76), (ii) the Global Ocean Data Analysis Project (Glodap; v2 2020; refs. 77,78), and (iii) satellite data (MODIS; refs. 79–81). For the downloaded WOA and GLODAP datasets we always extracted the deepest depth layer of each grid location. Based on the exact sampling coordinates we then extracted the datapoints of the closest positions present in the WOA and GLODAP bottom depth layer. The mixed layer depth data used in this study was derived from the NOAA Atlas NESDIS^82^, and the bathymetry data was based on ETOPO1. ETOPO1 relief data^83^ was also used as a basis for producing the world map in Fig. 1a. Correlations between the 24 environmental parameters were visualised with the help of a principal component analysis. In addition to the 24 continuous environmental parameters, we also analysed the following eight categorical environmental parameters: water mass, location ID, realm, ocean zone, world ocean (“parent” and “child”; with child providing higher resolution of parent), and geological setting (“parent” and “child”). These categorical parameters were standardised with the help of the following ontologies or frameworks: Location IDs of sponge grounds were standardised using Marine Regions Gazetteer, realm was based on the classification system by Costello and co-workers^84^, ocean zones were standardised according to the Environment Ontology (EnvO), world ocean was defined based on International Hydrographic Organisation (IHO) standards, and geologic setting was standardised according to the General Bathymetric Chart of the Oceans (GEBCO) framework and EnvO. All metadata were archived in the Pangaea database^85^.

### Reporting summary

Further information on research design is available in the Nature Research Reporting Summary linked to this article.

## Supplementary information

Supplementary Information

Supplementary Data

Reporting Summary

Description of Additional Supplementary Files

## Data Availability

The raw sequence data generated in this study (*16S*, *18S*, and *COI*) have been deposited within an Umbrella BioProject in the NCBI database under accession code PRJNA664762. SILVA data (version 138 SSU Ref NR 99) used to classify *16S* amplicon sequences is available at https://www.arb-silva.de/. Important intermediate outputs of processed *16S* data (i.e., ASV table and ASV taxonomy) were archived in the Zenodo database under accession code 10.5281/zenodo.6896034^86^. The ecological meta data and CTD profiles compiled in this study are available in the PANGAEA database under accession codes 10.1594/PANGAEA.923033^85^ and 10.1594/PANGAEA.923035^72^, respectively. In addition to our newly generated data, we used several publicly available resources to retrieve further data: the Word Ocean Atlas (version WOA18^73–76^) [https://www.ncei.noaa.gov], GLODAP (version v2 2020^77,78^) [https://www.ncei.noaa.gov], MODIS satellite data^79–81^ [https://oceandata.sci.gsfc.nasa.gov/], the ETOPO1 1 Arc-Minute Global Relief Model^83^ [https://www.ngdc.noaa.gov/mgg/global/], and the SMP dataset^9^ [10.1038/ncomms11870; Supplementary Data 2 of ref. 9. For data standardisation, Aphia IDs were retrieved from the World Register of Marine Species^70^ [https://www.marinespecies.org/]. Other data supporting the findings of this study are available within the article and its Supplementary Information and Supplementary Data files. Source data are provided with this paper.

## Source data

Source Data

## Code availability

*16S* amplicon pipeline laboratory procedures were archived at protocols.io (10.17504/protocols.io.kxygxer1kv8j/v1)^60^. Source code of the *16S* amplicon pipeline was archived at GitHub (https://kathrinbusch.github.io/16S-AmpliconCorePipeline/; 10.5281/zenodo.6857851)^61^.
