# Publisher Correction: Biodiversity, environmental drivers, and sustainability of the global deep-sea sponge microbiome

**DOI:** 10.1038/s41467-022-33966-7

**Published:** 2022-11-01

**Authors:** Kathrin Busch, Beate M. Slaby, Wolfgang Bach, Antje Boetius, Ina Clefsen, Ana Colaço, Marie Creemers, Javier Cristobo, Luisa Federwisch, Andre Franke, Asimenia Gavriilidou, Andrea Hethke, Ellen Kenchington, Furu Mienis, Sadie Mills, Ana Riesgo, Pilar Ríos, Emyr Martyn Roberts, Detmer Sipkema, Lucía Pita, Peter J. Schupp, Joana Xavier, Hans Tore Rapp, Ute Hentschel

**Affiliations:** 1 GEOMAR Helmholtz Centre for Ocean Research Kiel, Düsternbrooker Weg 20, 24105 Kiel, Germany; 2 MARUM-Center for Marine Environmental Sciences and Department of Geosciences, University of Bremen, 28359 Bremen, Germany; 3 MPI-Max Planck Institute for Marine Microbiology, Celsiusstr. 1, 28359 Bremen, Germany; 4 AWI-Alfred Wegener Institute Helmholtz Centre for Polar and Marine Research, Am Handelshafen 12, 27570 Bremerhaven, Germany; 5 OKEANOS-Institute of Marine Research, University of the Açores, Rua Prof Frederico Machado, 9901-862 Horta, Portugal; 6 MARBEC, University of Montpellier, CNRS, IFREMER, IRD, Avenue Jean Monnet, CS 30171 - 34203, Sète, France; 7 IEO-CSIC-Spanish Oceanographic Institute, Oceanographic Centre Gijón, Avda. Principe de Asturias 70 bis, 33212 Gijón, Spain; 8 University of Bremen, Faculty 2 Biology/Chemistry, Leobener Str., 28359 Bremen, Germany; 9 IKMB-Institute of Clinical Molecular Biology, Rosalind-Franklin-Straße 12, 24105 Kiel, Germany; 10 Wageningen University, Laboratory of Microbiology, Stippeneng 4, 6708WE Wageningen, the Netherlands; 11 DFO-Department of Fisheries and Oceans, Bedford Institute of Oceanography, P.O. Box 1006, 1 Challenger Dr., B2Y 4A2 Dartmouth, NS Canada; 12 NIOZ-Royal Netherlands Institute for Sea Research, 1790 AB Den Burg Texel, the Netherlands; 13 NIWA-National Institute of Water and Atmospheric Research, 301 Evans Bay Parade Hataitai, Wellington, New Zealand; 14 MNCN-National Museum of Natural Sciences, Department of Biodiversity and Evolutionary Biology, c/José Gutiérrez Abascal 2, 28006 Madrid, Spain; 15 NHM-Natural History Museum of London, Department of Life Sciences, Cromwell Road, SW7 5BD London, UK; 16 University of Bergen, Department of Biological Sciences and K.G. Jebsen Centre for Deep Sea Research, PO Box 7803, 5020 Bergen, Norway; 17 Bangor University, School of Ocean Sciences, Menai Bridge, LL59 5AB Anglesey, UK; 18 ICM-CSIC-Institute of Marine Sciences, Passeig de la Barceloneta 37-49, 08003 Barcelona, Spain; 19 ICBM-Institute for Chemistry and Biology of the Marine Environment, University of Oldenburg, Schleusenstraße 1, 26382 Wilhelmshaven, Germany; 20 HIFMB-Helmholtz Institute for Functional Marine Biodiversity, University of Oldenburg, Ammerländer Heerstraße 231, 26129 Oldenburg, Germany; 21 CIIMAR-Interdisciplinary Centre of Marine and Environmental Research, University of Porto, Avenida General Norton de Matos, S/N, 4450-208 Matosinhos, Portugal; 22 University of Kiel, Christian-Albrechts-Platz 4, 24118 Kiel, Germany

**Keywords:** Environmental microbiology, Microbial ecology, Symbiosis

Correction to: *Nature Communications* 10.1038/s41467-022-32684-4, published online 02 September 2022

The original version of the Description of Additional Supplementary Files associated with this Article contained errors in the legends of Supplementary Data 5–8 and omitted legends for the Source Data. The HTML has been updated to include a corrected version of the Description of Additional Supplementary Files; the original incorrect version of this file can be found as Supplementary Information associated with this Correction.

## Supplementary information

Description of Additional Supplementary Files

