# Supplementary material for: Biodiversity, environmental drivers, and sustainability of the global deep-sea sponge microbiome
Source: Nat Commun. 2022 Sep 2;13:5160. doi: 10.1038/s41467-022-32684-4 (PMC9440067; doi:10.1038/s41467-022-32684-4)
Supplement: Supplementary file 1 — Supplementary Information [file 41467_2022_32684_MOESM1_ESM.pdf]

Supplementary Information

to

Biodiversity, environmental drivers, and sustainability of the global deep-sea sponge microbiome

Kathrin Busch, Beate M. Slaby, Wolfgang Bach, Antje Boetius, Ina Clefsen, Ana Colaço, Marie Creemers, Javier Cristobo, Luisa Federwisch, Andre Franke, Asimenia Gavriilidou, Andrea Hethke, Ellen Kenchington, Furu Mienis, Sadie Mills, Ana Riesgo, Pilar Ríos, Emyr Martyn Roberts, Detmer Sipkema, Lucía Pita, Peter J. Schupp, Joana Xavier, Hans Tore Rapp, Ute Hentschel

This PDF file includes (click on blue text to jump to item):

1) **Supplementary Figures**.........2-14
   - Supplementary Figure 1
   - Supplementary Figure 2
   - Supplementary Figure 3
   - Supplementary Figure 4
   - Supplementary Figure 5
   - Supplementary Figure 6
   - Supplementary Figure 7
   - Supplementary Figure 8
   - Supplementary Figure 9
   - Supplementary Figure 10
   - Supplementary Figure 11
   - Supplementary Figure 12
   - Supplementary Figure 13
2) **Supplementary Tables**.........15-18
   - Supplementary Table 1
   - Supplementary Table 2
   - Supplementary Table 3
   - Supplementary Table 4
   - Supplementary Table 5
   - Supplementary Table 6
   - Supplementary Table 7
3) **Supplementary Note**.........19
   - Supplementary Note 1
4) **Supplementary Methods**.........20-22
   - Supplementary Methods 1
   - Supplementary Methods 2
5) **Supplementary References**.........23-24

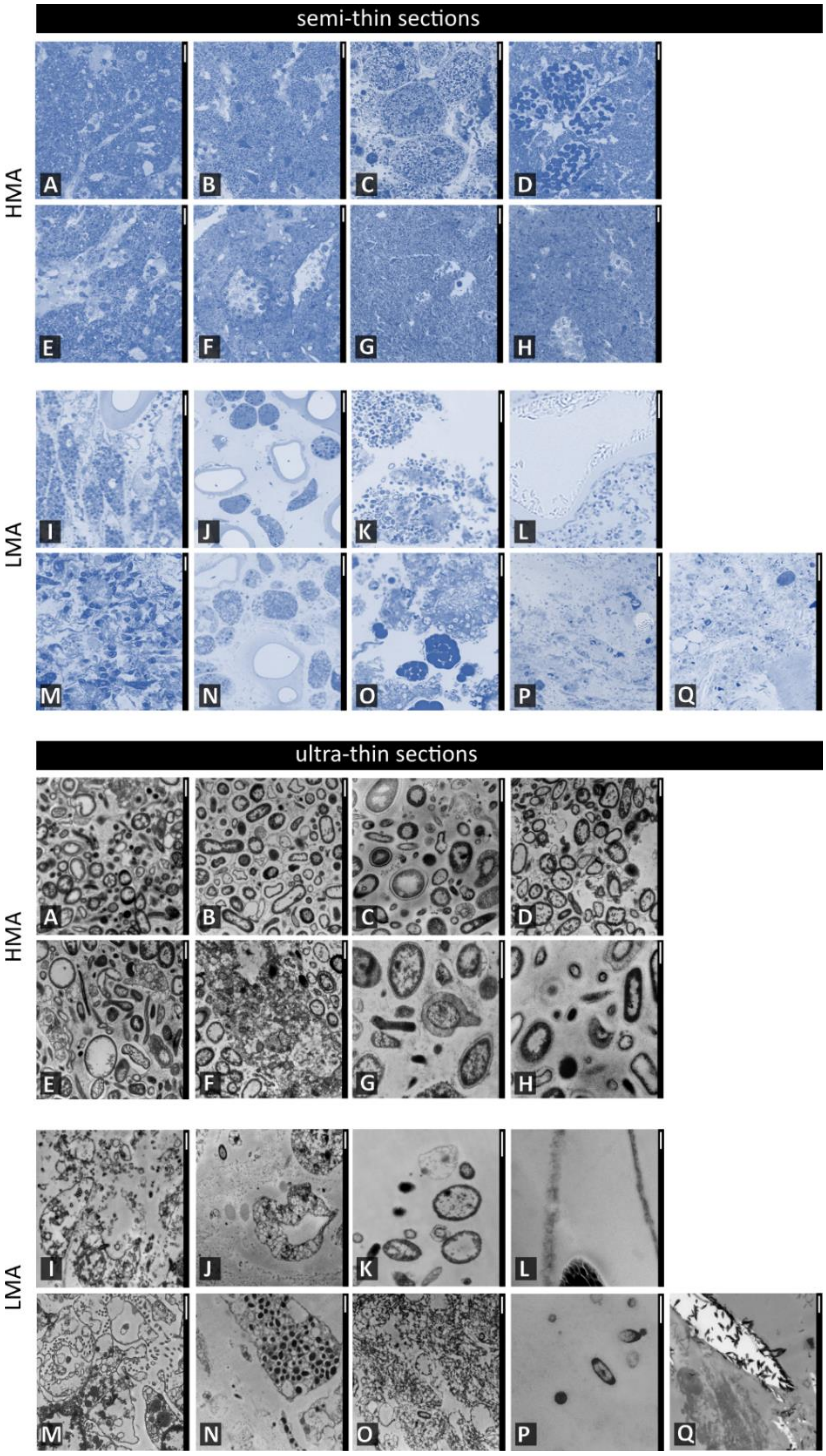

Supplementary Figure 1 Representative micrographs showing microbial cells within the tissues of 17 selected sponge species. For each of the 17 selected sponge species three biological replicates were processed, and at least ten random tissue areas were inspected in each sample to confirm representativeness of the shown selected micrographs. Upper panel shows semi-thin sections (scale bars= 10 µm) and lower panel shows transmission electron microscopy of ultra-thin sections (scale bars= 1 µm). In each panel, High Microbial Abundance (HMA) sponges are shown above (a-h) and Low Microbial Abundance (LMA) sponges are shown below (i-q). **a)** *Geodia parva* **b)** *Geodia phlegraei* **c)** *Petrosia crassa* **d)** *Stryphnus fortis* **e)** *Geodia hentscheli* **f)** *Plakortis simplex* **g)** *Craniella infrequens* **h)** *Stelletta rhaphidiophora* **i)** *Axinella infundibuliformis* **j)** *Phakellia ventilabrum* **k)** *Vazella pourtalesii* **l)** *Aphrocallistes beatrix* **m)** *Acantheurypon spinispinosum* **n)** *Phakellia hirondellei* **o)** *Schaudinnia rosea* **p)** *Thenea schmidti* **q)** *Pheronema carpenteri*.

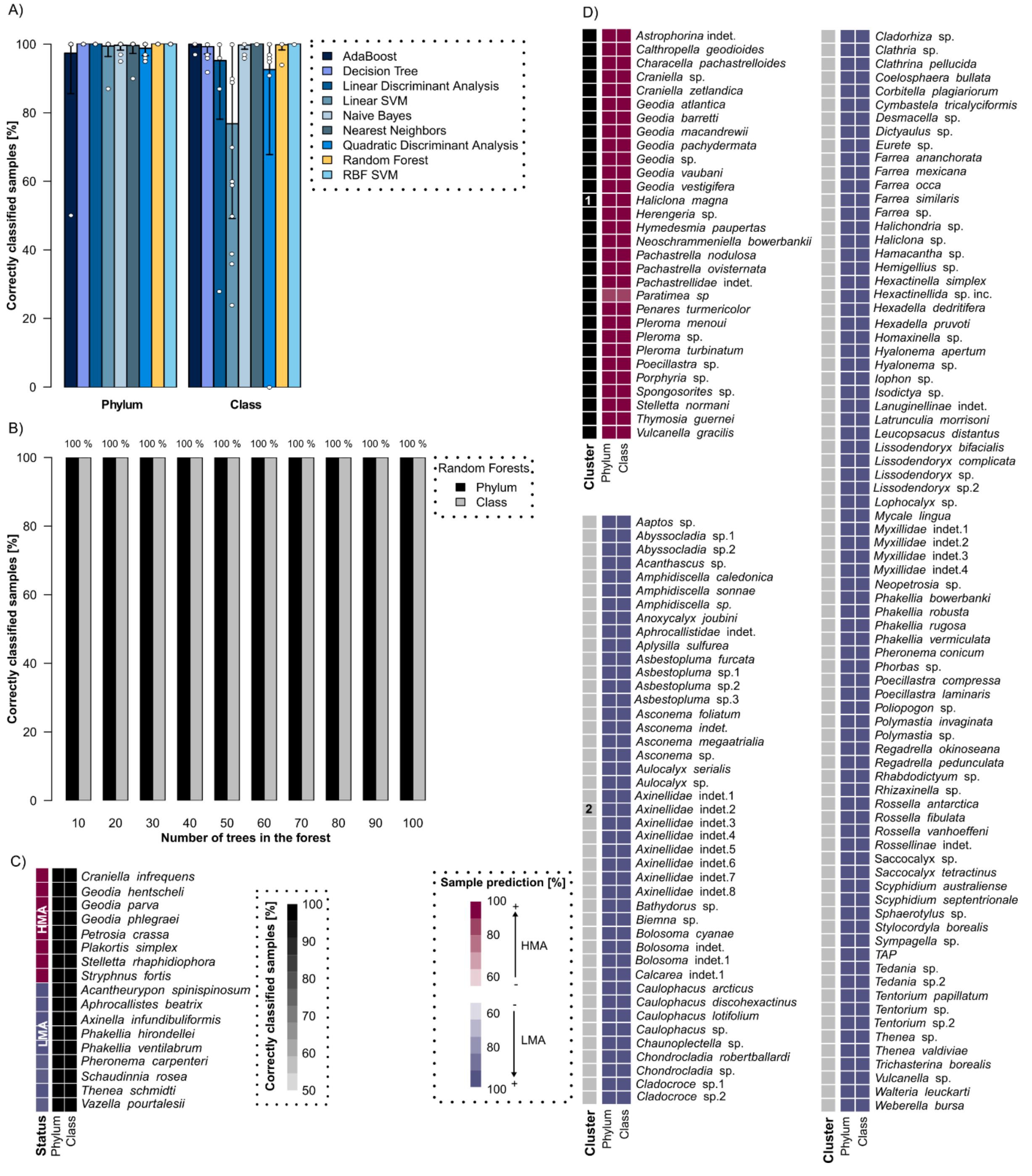

Supplementary Figure 2 Visual outputs of a machine learning approach (after:[1]) to predict the HMA-LMA status of 169 sponge species. a-c) Selection and standardisation of classifiers: **a)** Comparing the performance of different algorithms and taxonomic levels on the training dataset (i.e. sponge species with HMA-LMA status determined by microscopy). Bars show mean values across the training dataset (n=17 sponge species in the training dataset). Error bars represent weighted standard deviations. White dots indicate raw data points as overlay. The overall mean of correctly classified samples is 99.3% on phylum level, and 95.9% on class level (n=153 classifications). Source data are provided as a Source Data file. **b)** Performance of Random Forests in relation to number of trees in the forest. Percentages above the bars represent means of weighted averages. **c)** Performance of Random Forests algorithm in predicting the HMA-LMA status of sponge species with known status. **d)** Predictions of the HMA-LMA status of previously uncharacterised sponge species by the Random Forests algorithm. The applied number of trees in the forest was 50 in c) and d).

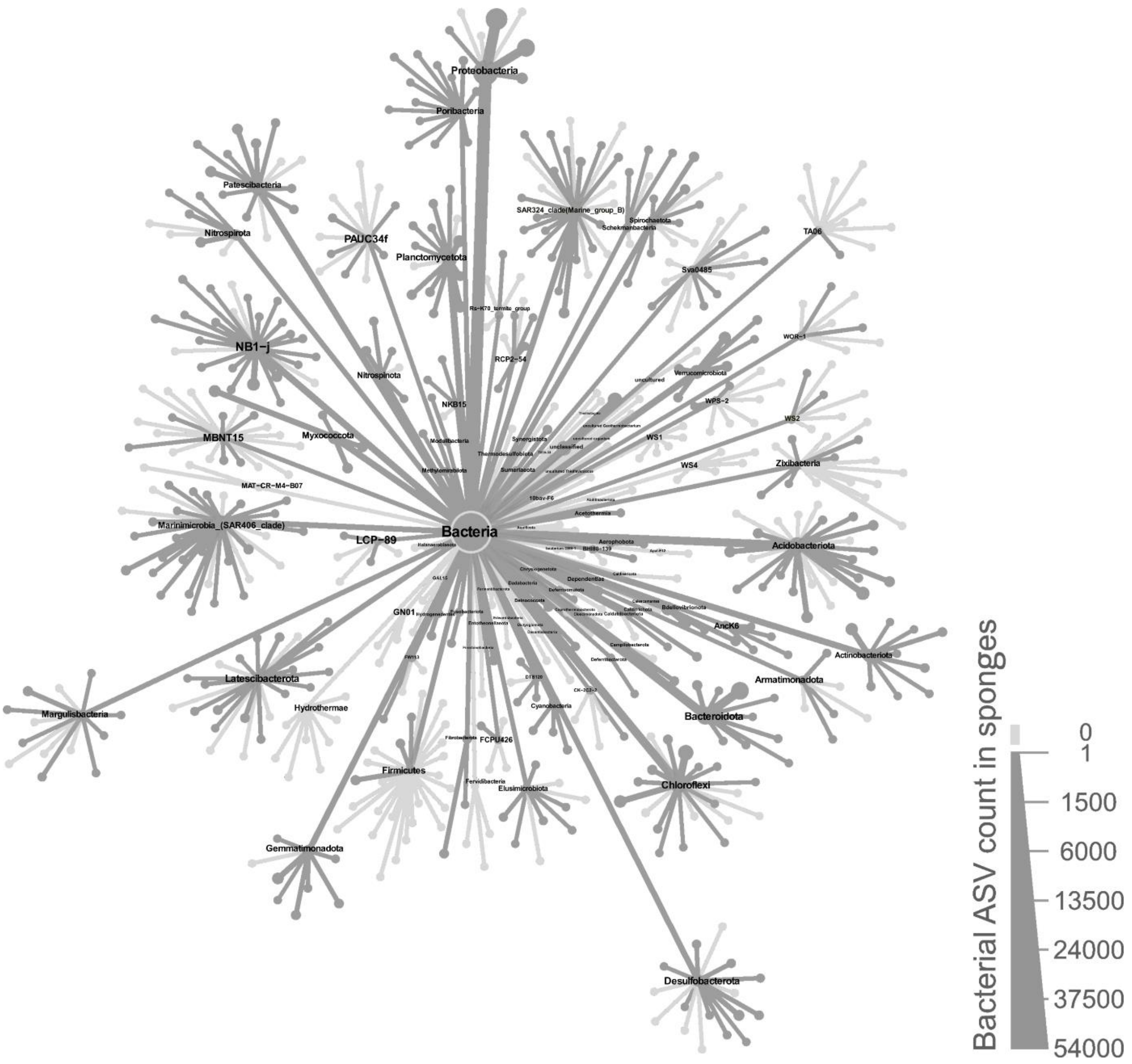

Supplementary Figure 3 Heat tree showing microbial taxon richness found in the deep-sea sponge collection (n=931). Bacterial phyla are split up into bacterial classes and are shown in alphabetical order. Those phyla and classes found in sponges are colored in dark grey. The current SILVA database (version 138) served as taxonomic backbone for all currently known bacterial taxa (light grey). Sizes of nodes and lines encode for the bacterial ASV richness behind each phylum and class.

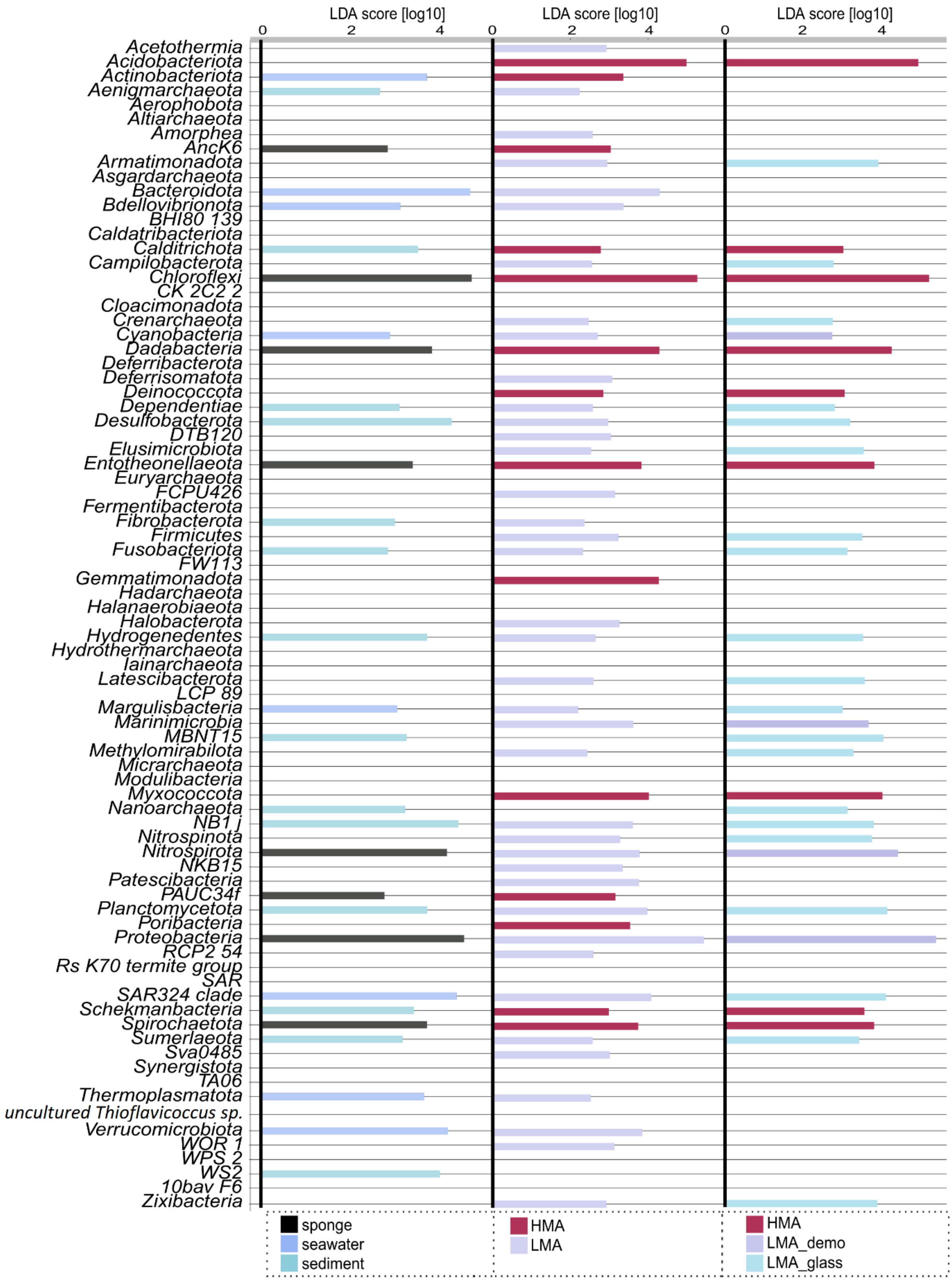

Supplementary Figure 4 Linear discriminant analysis (LDA) Effect Size (LEfSe) plots. All detected microbial phyla are shown in alphabetical order on the y-axis. Colored bars are shown if phyla were significantly enriched in one sample category. Left most panel shows differences between sponge, seawater, and sediment. Middle panel shows differences between HMA and LMA sponges, and the right most panel shows differences between HMA, LMA_demo, and LMA_glass sponges.

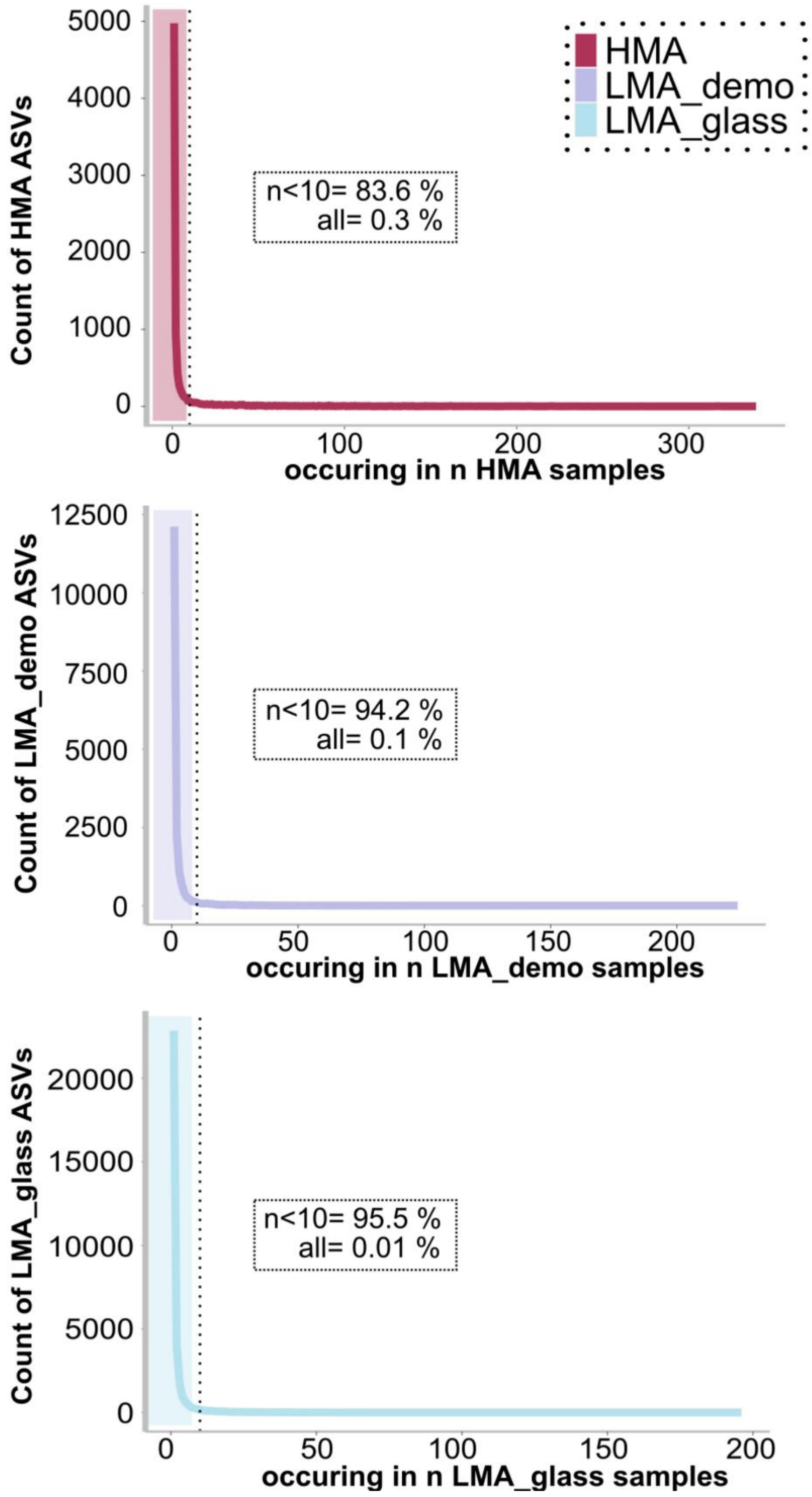

Supplementary Figure 5 Distribution of ASV counts across sponge samples in the three categories. The dotted vertical line marks 10 sponge samples. The majority of ASVs (≥ 83.6%) occurs in less than 10 sponge samples while a small fraction (≤ 0.3%) occurs in all samples.

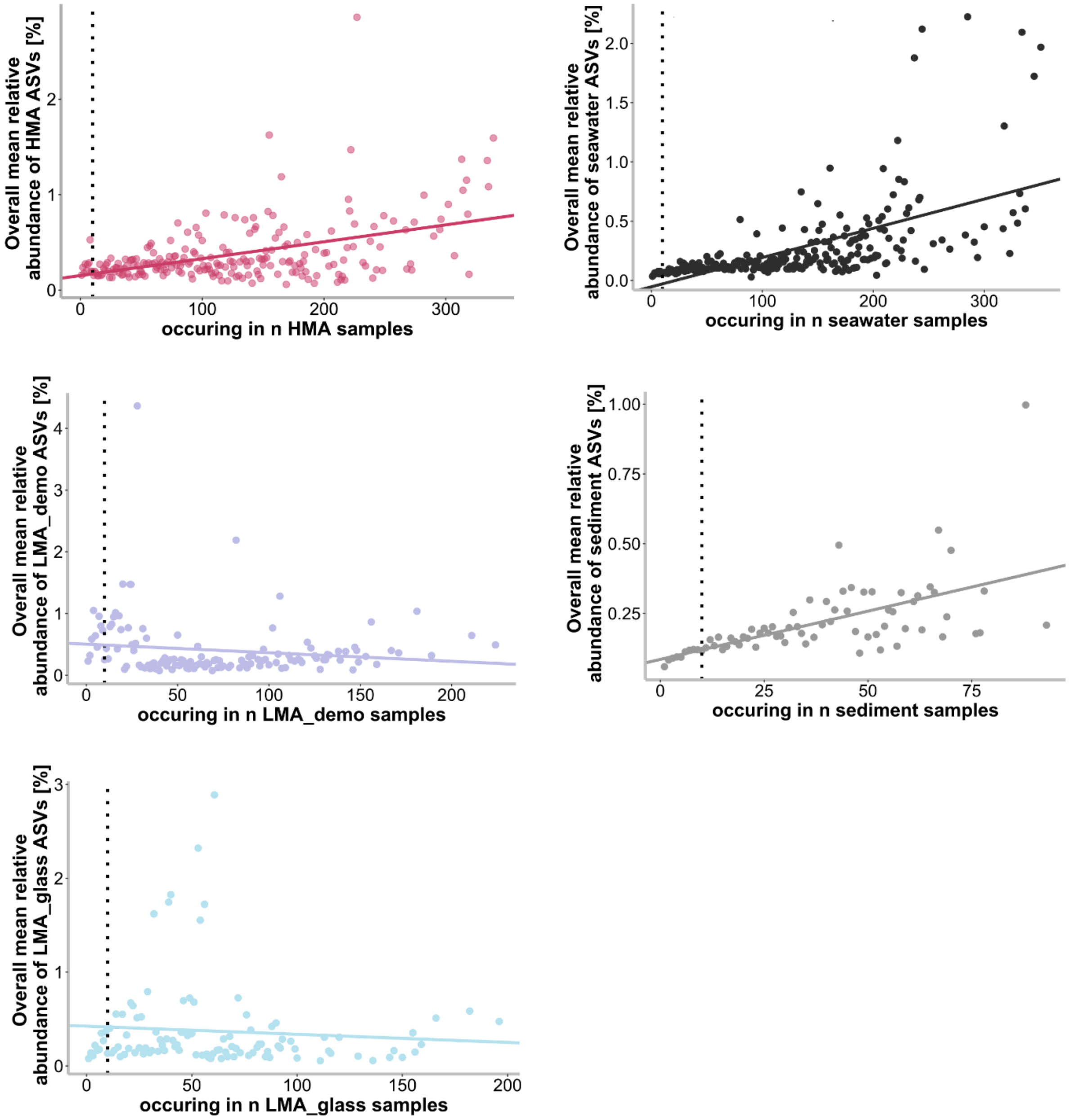

Supplementary Figure 6 Mean relative ASV abundances presented in relation to the number of samples in which the respective ASVs occur in the three sponge types and environmental reference samples. The dotted vertical line marks 10 samples.

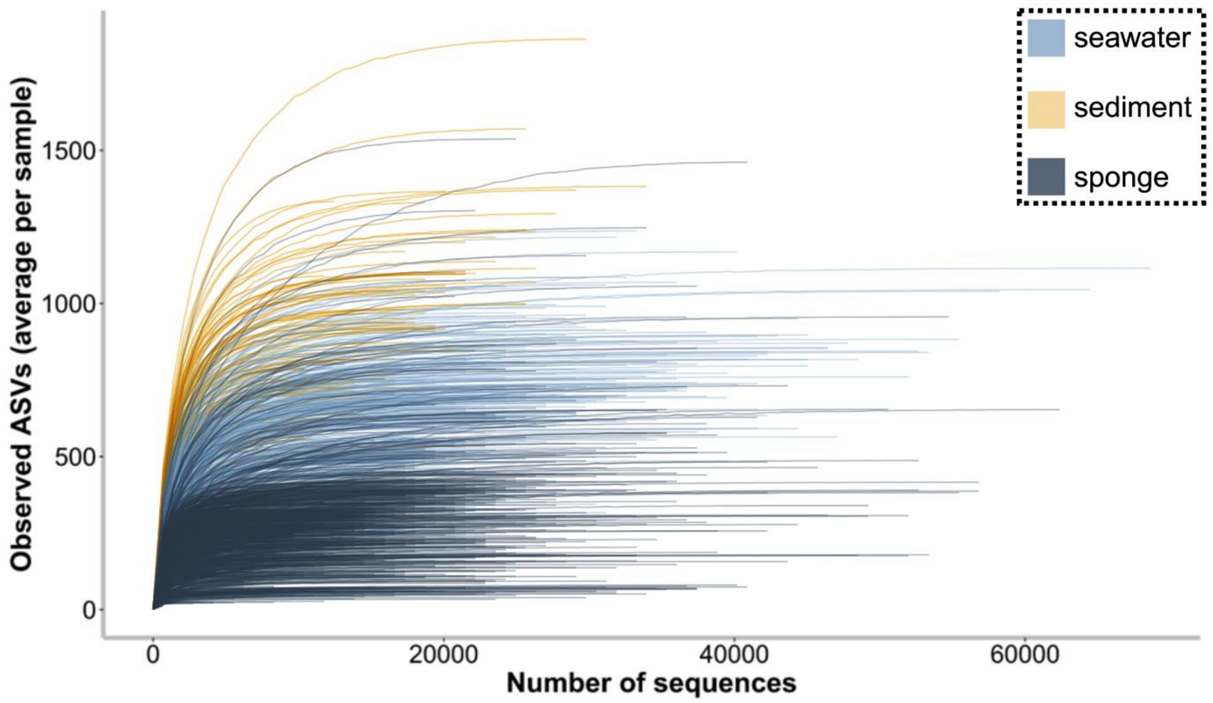

Supplementary Figure 7 Richness of individual samples from microbial communities in seawater, sediments and sponges. Rarefaction curves of *16S* rRNA gene diversity are shown for seawater (light blue), sediment (yellow) and sponge (dark blue) samples.

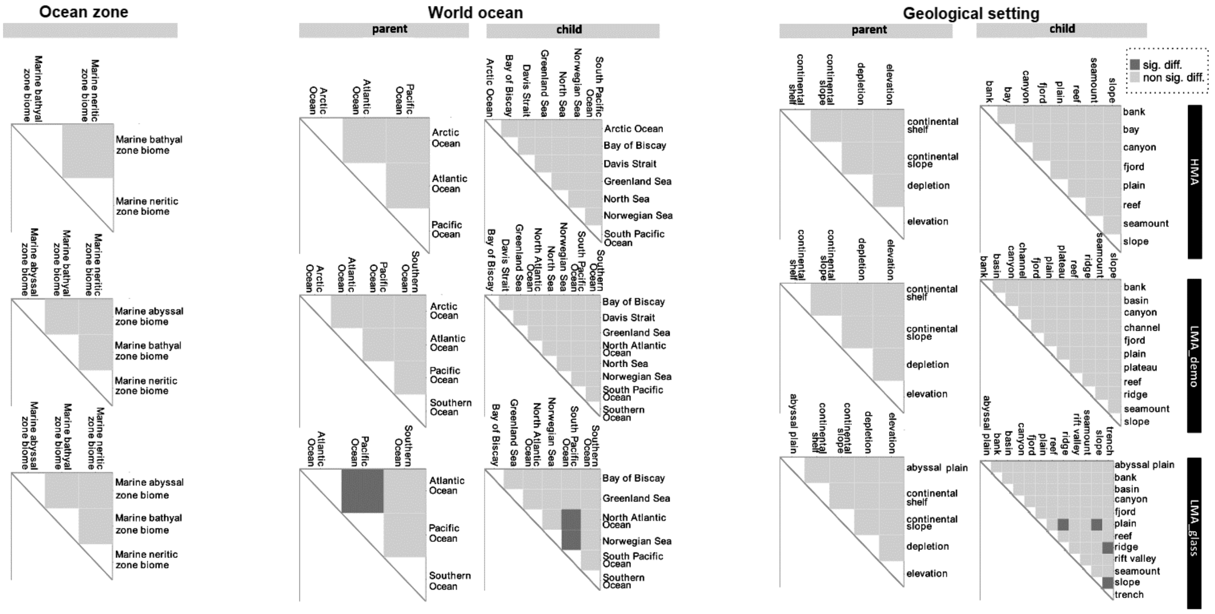

Supplementary Figure 8 Visual representation of statistical testing results (Dunn's tests) conducted to assess variations in microbial alpha-diversity (Shannon index) between ocean zones (standardised according to ENVO), world ocean (standardised according to IHO), and geological setting (standardised according to GEBCO and ENVO). "Child" column provides higher resolution for "parent" column.

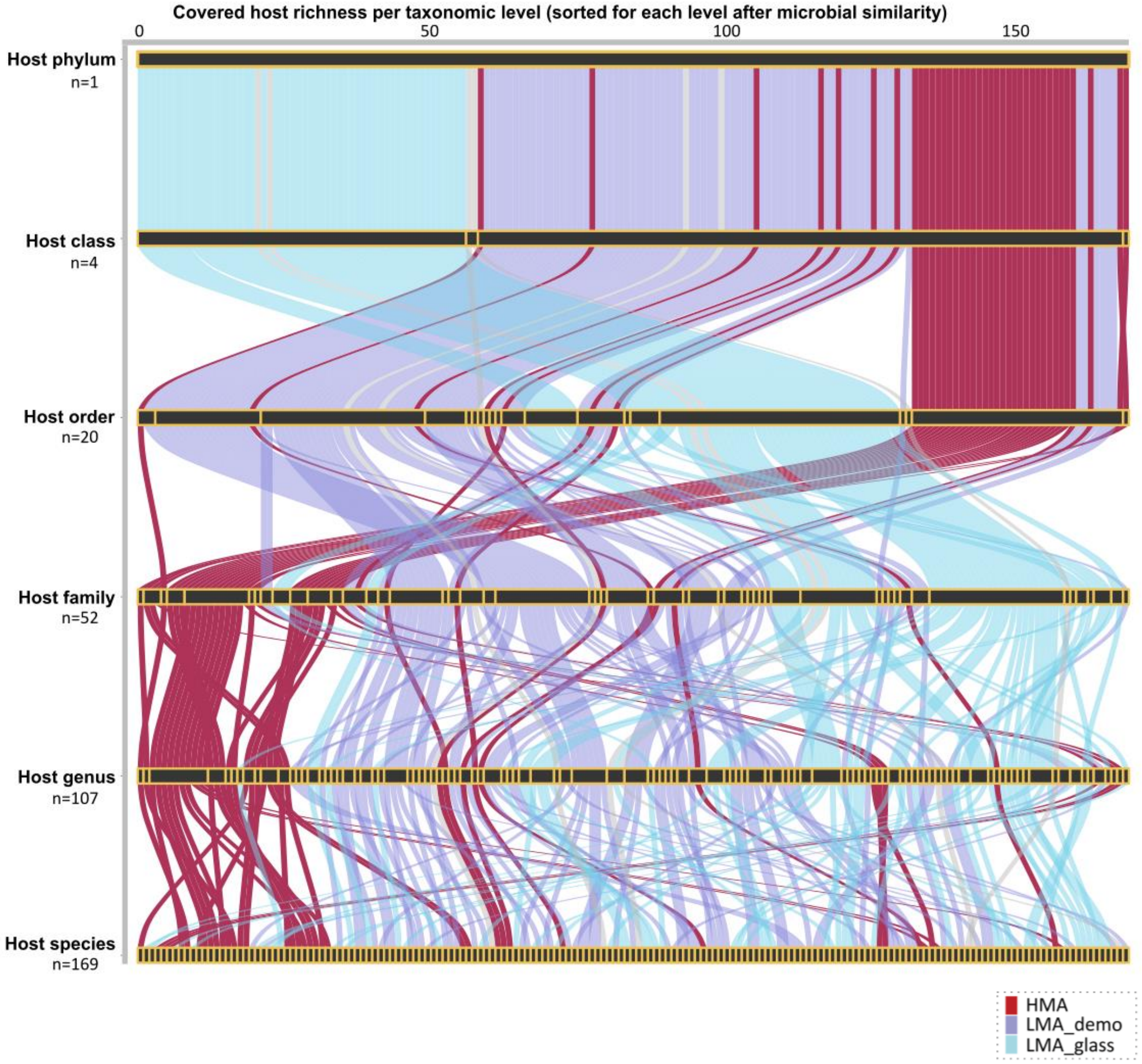

Supplementary Figure 9 Microbial beta-diversity across different sponge host taxonomic levels. Alluvial diagram showing clustering based on microbial community similarity (weighted UniFrac distances) at different host taxonomic levels. Yellow lines mark boundaries of sponges belonging to the same taxonomic group. Colors are added according to sponge type (red= HMA, dark blue = LMA_demo, light blue = LMA_glass).

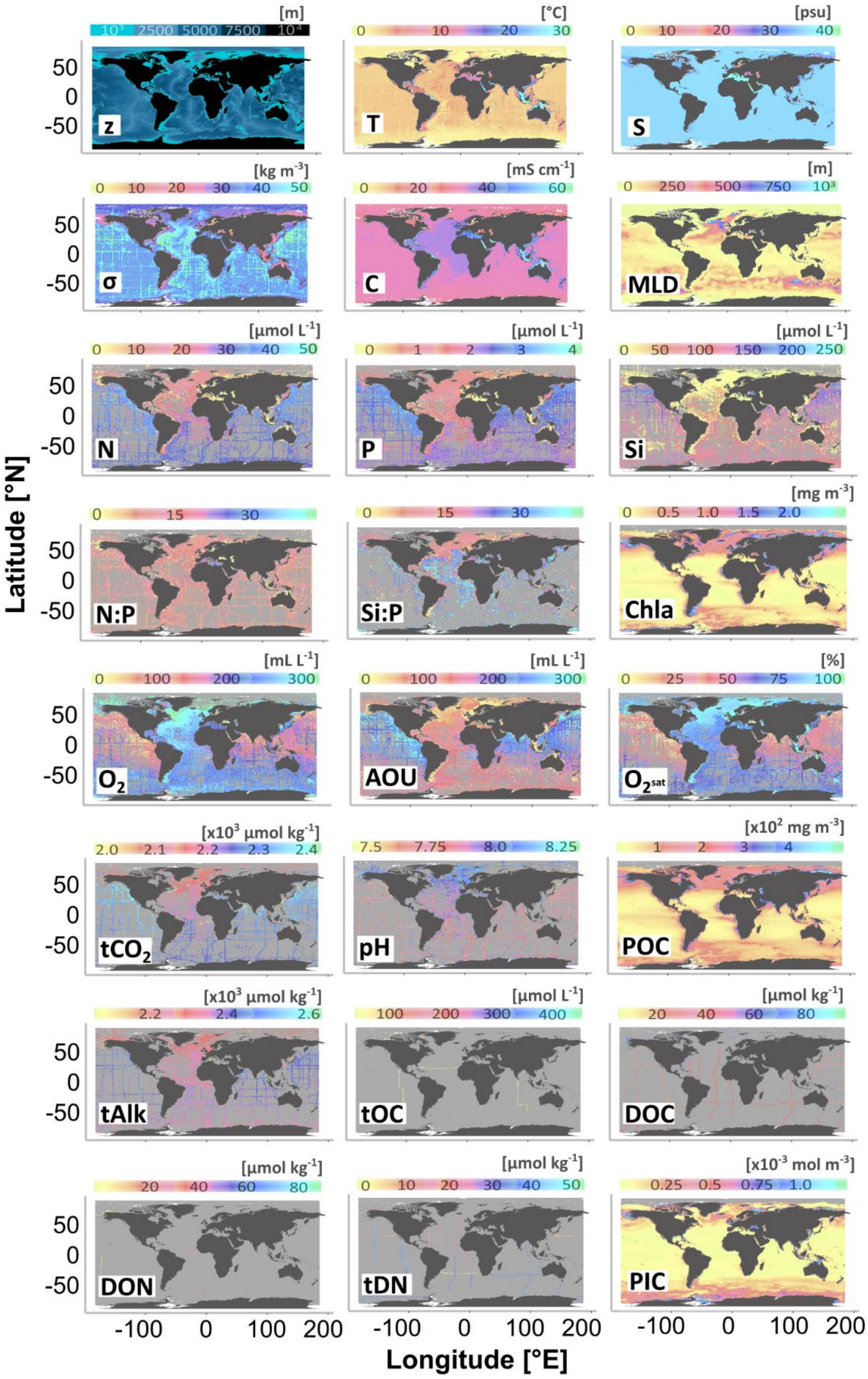

Supplementary Figure 10 Characteristics of physical and biogeochemical parameters at the seafloor on a global scale. Data were compiled based on the following climatologies and datasets: World Ocean Atlas (WOA; version WOA18), Global Ocean Data Analysis Project (Glodap; v2 2020), satellite data (MODIS), and ETOPO1 bathymetry.

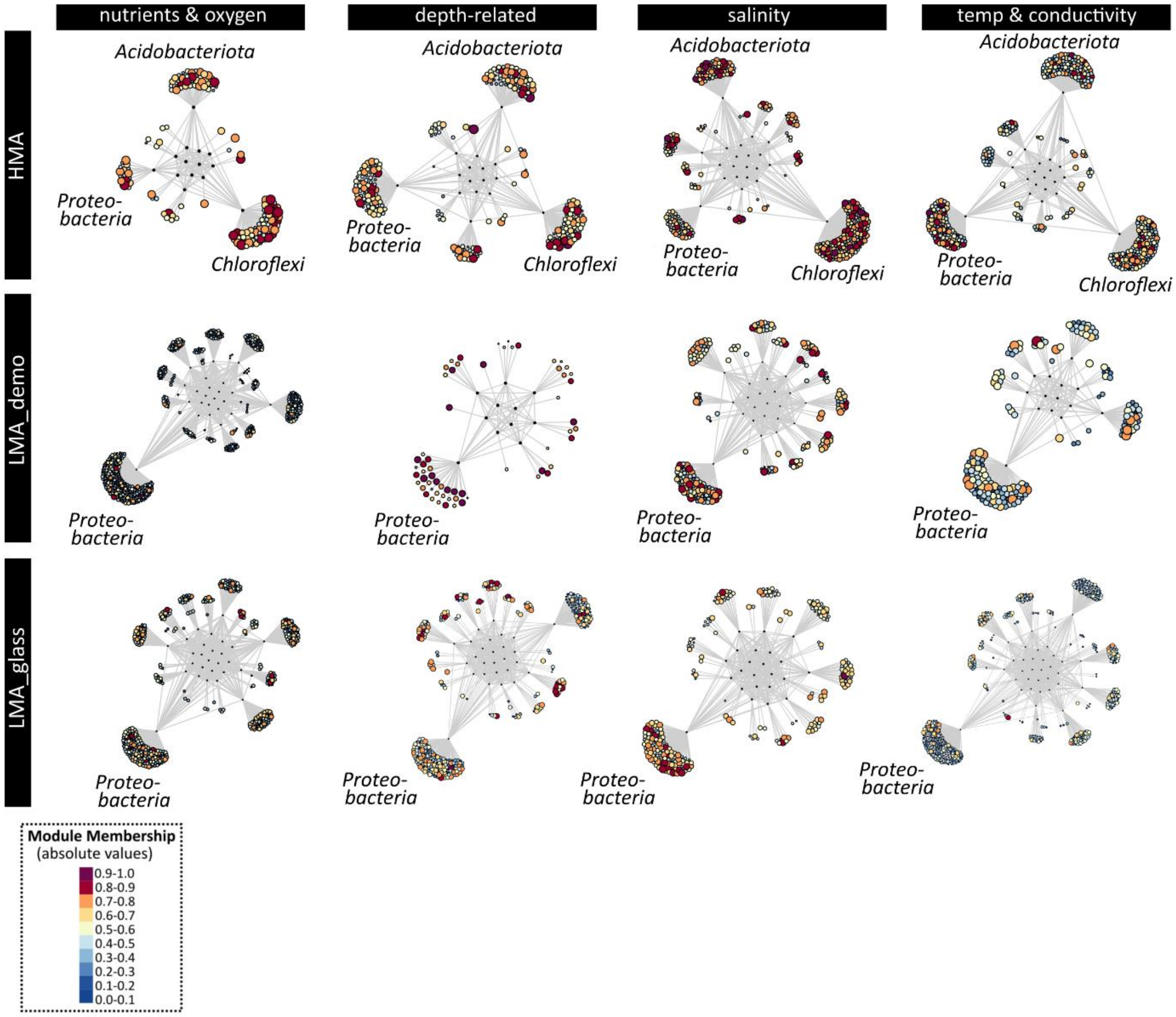

Supplementary Figure 11 Network visualisation of microbial community compositions occurring in the environmental parameter modules (nutrients & oxygen, depth-related, salinity, and temperature modules) of HMA sponges, LMA_demo sponges, and LMA_glass sponges. Modules were derived from weighted gene correlation networks. Colors and node sizes in the network indicate modularity of respective microbial taxa. Modularity indicates for each taxon the degree of connections to other taxa in the network as well as the correlation to the respective environmental parameter. Only the most abundant taxa are labelled.

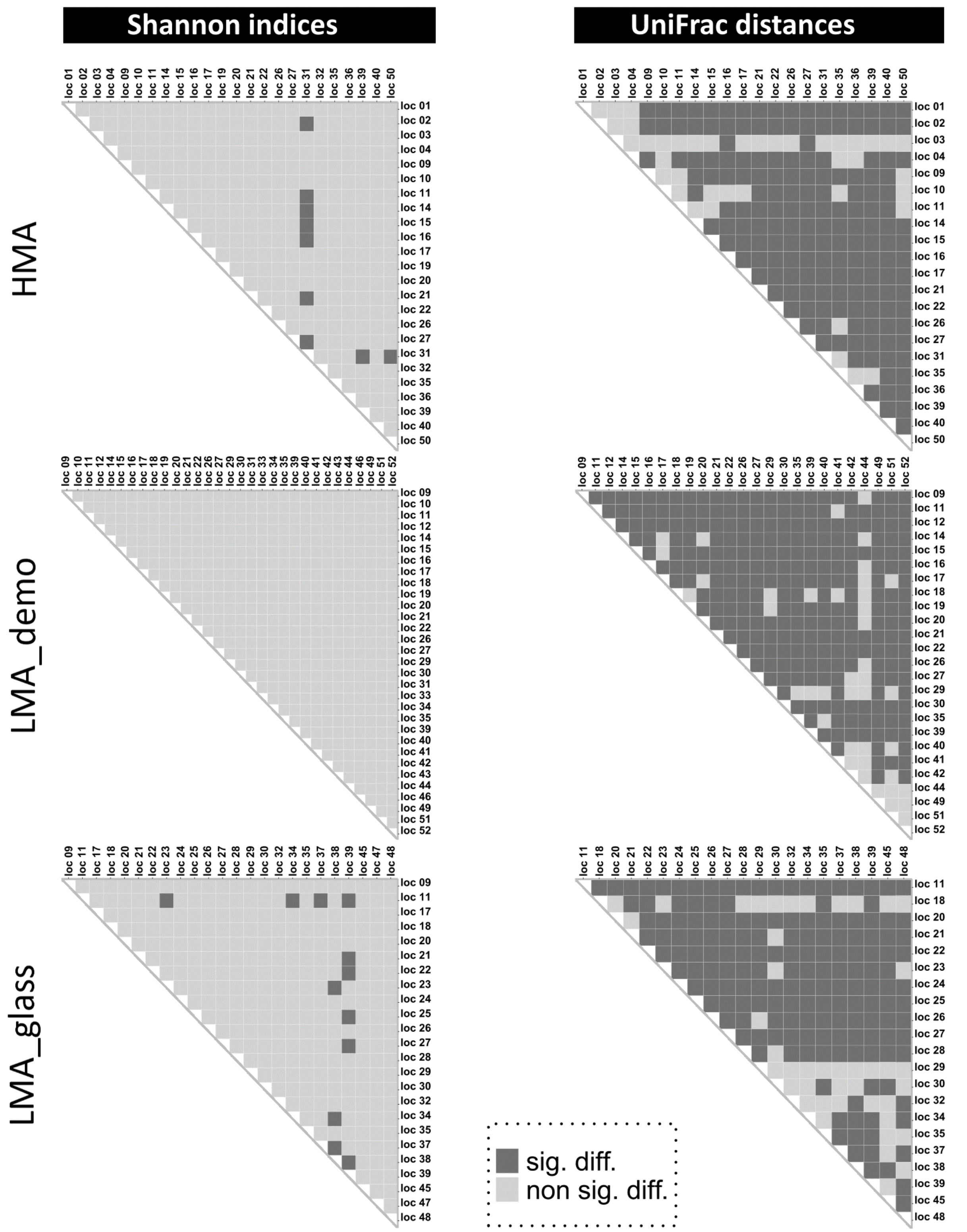

Supplementary Figure 12 Visual representation of statistical testing results (Dunn's tests) conducted to assess variations in microbial alpha-diversity (Shannon index) between geographic locations (left panel) and results of statistical testing (PERMANOVAs based on weighted UniFrac distances) to assess differences in the microbial community composition between geographic locations (right panel). The exact values of each pairwise test statistics are found in **Supplementary Data 6** and **Supplementary Data 7**.

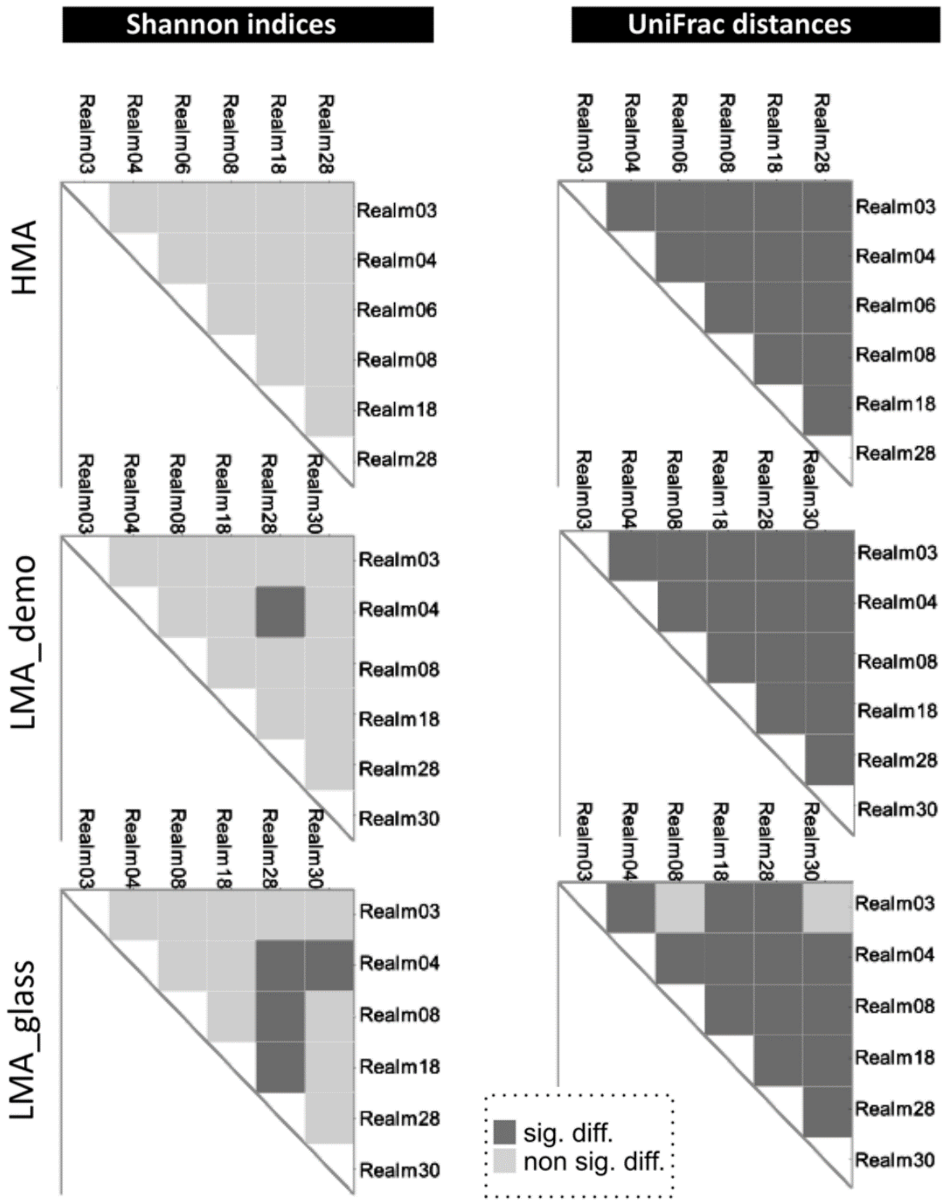

Supplementary Figure 13 Visual representation of statistical testing results (Dunn's tests) conducted to assess variations in microbial alpha-diversity (Shannon index) between realms (left panel; according to [2]) and results of statistical testing (PERMANOVAs based on weighted UniFrac distances) to assess differences in the microbial community composition between realms (right panel). The exact values of each pairwise test statistics are found in **Supplementary Data 8** and **Supplementary Data 9**.

Supplementary Table 1 Key data for the 21 ship expeditions conducted in the years 2012-2019. Cruise leg refers to the official campaign name that is used in public repositories (PANGAEA).

	Cruise leg	Cruise platform	Departure date	Departure location	Return date	Return location
**1**	Hudson2016-019	CCGS Hudson	2016-07-19	Dartmouth (Canada)	2016-08-16	Dartmouth (Canada)
**2**	PS96 (ANT XXXI/2)	RV Polarstern	2015-12-06	Cape Town (South Africa)	2016-02-14	Punta Arenas (Chile)
**3**	SO254	RV Sonne	2017-01-27	Auckland (New Zealand)	2017-02-26	Auckland (New Zealand)
**4**	PS80 (ARK-XXVII/2)	RV Polarstern	2012-07-15	Longyearbyen (Spitsbergen)	2012-07-30	Tromsø (Norway)
**5**	PS107 (ARK-XXXI/2)	RV Polarstern	2017-07-23	Tromsø (Norway)	2017-08-19	Tromsø (Norway)
**6**	KB2017610	RV Kristine Bonnevie	2017-04-26	Bergen (Norway)	2017-05-02	Bergen (Norway)
**7**	MLB2017001	CCGS Martha L. Black	2017-08-31	Sydney (Canada)	2017-09-07	Dartmouth (Canada)
**8**	GS2016109A	RV G.O.Sars	2016-06-18	Tromsø (Norway)	2016-06-27	Tromsø (Norway)
**9**	GS2017110	RV G.O.Sars	2017-07-20	Bergen (Norway)	2017-08-06	Tromsø (Norway)
**10**	SPONGES 0617	B/O Ángeles Alvariño	2017-06-11	Gijón (Spain)	2017-06-25	Gijón (Spain)
**11**	HB2016952	RV Hans Brattstrøm	2016-09-08	Marineholmen (Norway)	2016-09-09	Marineholmen (Norway)
**12**	CV13012	RV Celtic Voyager	2013-08-20	Galway (Ireland)	2013-08-27	Galway (Ireland)
**13**	0915S	FRV Scotia	2015-07-16	Aberdeen (UK)	2015-07-27	Aberdeen (UK)
**14**	HB27102017	RV Hans Brattstrøm	2017-10-27	Bergen (Norway)	2017-10-27	Bergen (Norway)
**15**	JR17003a	RRS James Clark Ross	2018-02-18	Stanley (Falkland Islands)	2018-03-12	Stanley (Falkland Islands)
**16**	PAA2014007	GINR Paamiut	2014-09-22	Nuuk (Greenland)	2014-10-19	Nuuk (Greenland)
**17**	GS2018108	RV G.O.Sars	2018-07-28	Tromsø (Norway)	2018-08-14	Tromsø (Norway)
**18**	Azores2018	RV Ada Rebikoff	2018-07-04	Pico (Azores)	2018-07-11	Pico (Azores)
**19**	PS101 (ARK-XXX/3)	RV Polarstern	2016-09-09	Tromsø (Norway)	2016-10-23	Bremerhaven (Germany)
**20**	MSM86	RV Maria S. Merian	2019-08-14	Longyearbyen (Spitsbergen)	2019-09-17	Emden (Germany)
**21**	H045	fishing vessel	2018-05	NA	2018-05	NA

Supplementary Table 2 Dunn's statistical testing to assess variations in microbial alpha-diversity (Shannon index) between sample types. The tests were run two-sided and with Bonferroni corrections.

Group1	Group2	Sample number	Dunns Z	p-value
seawater	sponge	1286	21.49	<0.001
sediment	sponge	1039	16.75	<0.001
seawater	sediment	463	-14.19	<0.001
HMA	LMA_demo	670	21.82	<0.001
HMA	LMA_glass	602	18.42	<0.001
LMA_demo	LMA_glass	554	-1.98	0.048

Supplementary Table 3 Two-sided PERMANOVAs based statistical testing on weighted UniFrac distances to assess differences in the microbial community composition between sample types.

Group1	Group2	Sample number	Permutations	pseudo F	p-value
seawater	sponge	1286	999	257.6	0.001
sediment	sponge	1039	999	62.1	0.001
seawater	sediment	463	999	205.5	0.001
HMA	LMA_demo	670	999	668.5	0.001
HMA	LMA_glass	602	999	515.9	0.001
LMA_demo	LMA_glass	554	999	65.8	0.001

Supplementary Table 4 Unclassified microbial taxa in deep-sea sponges that were compiled on different microbial taxonomic levels.

Level	Count of unclassified ASVs	Fraction unclassified ASVs of total ASV count [%]	Relative abundance unclassified ASVs in average community [%]
phylum	2484	4.6	2.4
class	5536	10.3	4.7
order	15025	28.0	19.9
family	23904	44.5	50.4
genus	34115	63.5	71.9

Supplementary Table 5 Fractions of amplicon sequence variants (ASVs) shared between different sample types. Values are percentages [%] calculated based on the total number of ASVs present in the two compared groups.

HMA	LMA_demo	LMA_glass	seawater	sediment	
-	4.3	2.8	1.8	1.4	**HMA**
	-	12.4	14.6	5.4	**LMA_demo**
		-	16.2	12.1	**LMA_glass**
			-	7.2	**seawater**
				-	**sediment**

Supplementary Table 6 Abbreviations of the 24 continuous environmental parameters and their full names and units. Method refers to the approach with which the data were gathered. *In situ* means that the respective data entries were measured during the 21 conducted cruises. WOA, MODIS, and GLODAP indicate that entries were retrieved from publicly available climatologies. In particular the World Ocean Atlas (WOA; version WOA18 [3–6]), the Global Ocean Data Analysis Project (Glodap; v2 2020 [7-8]), and satellite data (MODIS [9–11]) were used.

Abbreviation	Full parameter name [unit]	Method
**AOU**	Apparent oxygen utilisation at sampling depth [mL L^{-1}]	WOA
**Chla**	Chlorophyll a concentration at ocean surface [mg m^{-3}]	MODIS
**cond**	Conductivity of seawater at sampling depth [mS cm^{-1}]	*in situ*
**dens**	Density of seawater at sampling depth [kg m^{-3}]]	*in situ*
**depth**	Sampling depth [m]	*in situ*
**DOC**	Dissolved organic carbon concentration at sampling depth [µmol kg^{-1}]	GLODAP
**lat**	Sampling position, latitude [dd]	*in situ*
**lon**	Sampling position, longitude [dd]	*in situ*
**MLD**	Distance from sampling depth to mixed layer depth [m]	calculated
**N**	Nitrate concentration at sampling depth [µmol L^{-1}]	WOA
**N:P**	N:P ratio at sampling depth	calculated
**O2**	Oxygen concentration at sampling depth [mL L^{-1}]	WOA
**O2sat**	Oxygen saturation at sampling depth [%]	WOA
**P**	Phosphate concentration at sampling depth [µmol L^{-1}]	WOA
**pH**	pH at sampling depth	GLODAP
**PIC**	Particulate inorganic carbon concentration at ocean surface [mol m^{-3}]	MODIS
**POC**	Particulate organic carbon concentration at ocean surface [mg m^{-3}]	MODIS
**pres**	Pressure at sampling depth [dbar]	*in situ*
**S**	Salinity at sampling depth [psu]	*in situ*
**Si**	Silicate concentration at sampling depth [µmol L^{-1}]	WOA
**Si:P**	Si:P ratio at sampling depth	calculated
**T**	Temperature at sampling depth [°C]	*in situ*
**tAlk**	Total alkalinity of seawater at sampling depth [µmol kg^{-1}]	GLODAP
**tCO2**	Total dissolved carbon in seawater at sampling depth [µmol kg^{-1}]	GLODAP

Supplementary Table 7 List of all 25 water mass acronyms and their full names, determined manually based on literature[12-24]. The 14 water masses from which biological samples were obtained are marked by asterisks.

Acronym		Water mass
**AAIW**	*	Antarctic Intermediate Water
**AASW**		Antarctic Surface Water
**ADW**	*	Arctic Deep Water
**AIW**	*	Arctic Intermediate Water
**ASW**		Arctic Surface Water
**AW**	*	Atlantic Water
**BTW**		Brackish Top Water
**CBS-CIL**		Cabot Strait Surface - Cold Intermediate Layer
**CBSS**		Cabot Strait Sub-Surface Water
**CDW**	*	Circumpolar Deep Water
**ENACW**	*	Eastern North Atlantic Central Water
**ESW**	*	Eastern Shelf Water
**InLC**		Inshore Labrador Current
**LSW**	*	Labrador Sea Water
**MNwCCW**	*	Modified Norwegian Coastal Current Water
**MOW**	*	Mediterranean Outflow Water
**NwCCW**	*	Norwegian Coastal Current Water
**PW**		Polar Water
**SAW**		Sub-Antarctic Water
**SPSTMW**	*	South Pacific Sub-Tropical Mode Water
**STW**		Sub-Tropical Water
**TW**		Tropical surface Water
**WSPW**	*	Western South Pacific Water
**WSW**	*	Warm Slope Water
**WW**		Winter water

Supplementary Note 1

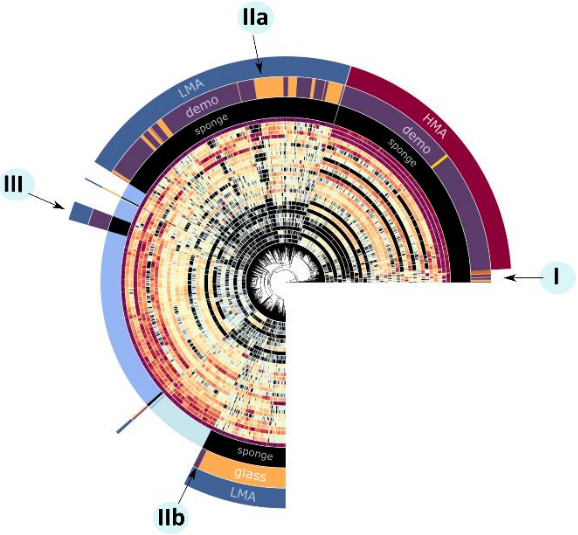

Several sponge groups showed deviations from the observed microbial alpha- and beta-diversity patterns. In the following these “outlier” sponge groups (n= min 3 and max 39 individuals per species) are discussed (see also **Figure 1b** and **Figure 3d**).

(I): The sponge species *Paratimea* sp., *Coelosphaera bullata*, *Myxillidae* indet.4 showed atypical microbiome profiles compared to the standard HMA-LMA sponges and thus fell outside of the grouping. *Paratimea* sp. sponges contained a particularly high abundance of Poribacteria (8.3%). *Coelosphaera bullata* sponges had a particularly high fraction of unclassified taxa (29.5% on phylum level) and of SAR324 clade bacteria (24.4%). *Myxillidae* indet.4 sponges had a reduced relative amount of Acidobacteriota (0.04%) and other phyla, as well as a higher relative amount of unclassified ASVs (2.9% on phylum level) in comparison to the standard HMA profile. The glass sponge species *Amphidiscella caledonica* (belonging to the family Euplectellidae) was distinct from the typical HMA-LMA profiles in that Chloroflexi were particularly abundant (63.3%) in this sponge species.

Sponges of the class Calcarea had a distinct microbiome, which clustered apart from demosponges and glass sponges. Sponges of the class Homoscleromorpha clustered together with demosponges.

(IIa): Some LMA glass sponge microbiomes clustered with those from LMA demosponges (see also **Figure 1b**). These were the glass sponge family *Euplectellidae* including the species *Amphidiscella* sp., *Bolosoma cyanae, Corbitella plagiariorum*, *Regadrella okinoseana*, *Regadrella pedunculata*, and *Saccocalyx tetractinus*. Furthermore, the microbiomes of all glass sponges belonging to the order Amphidiscosida, to the family Farreidae, and the sponge species *Aphrocallistes beatrix* and *Scyphidium australiense* clustered with LMA demosponges. (IIb): Conversely, the microbiomes of two LMA demosponges *Halichondria* sp. and *Phakellia bowerbanki* clustered with those from LMA glass sponges.

(III): The microbial alpha-diversity of *Aulocalyx* sp., *Leucopsacus distantus*, *Axinellidae* indet.4, and all individuals belonging to the TAP clade was most similar to that of seawater (20.1% overlap of ASVs between seawater and sponges, **Supplementary Table 5**).

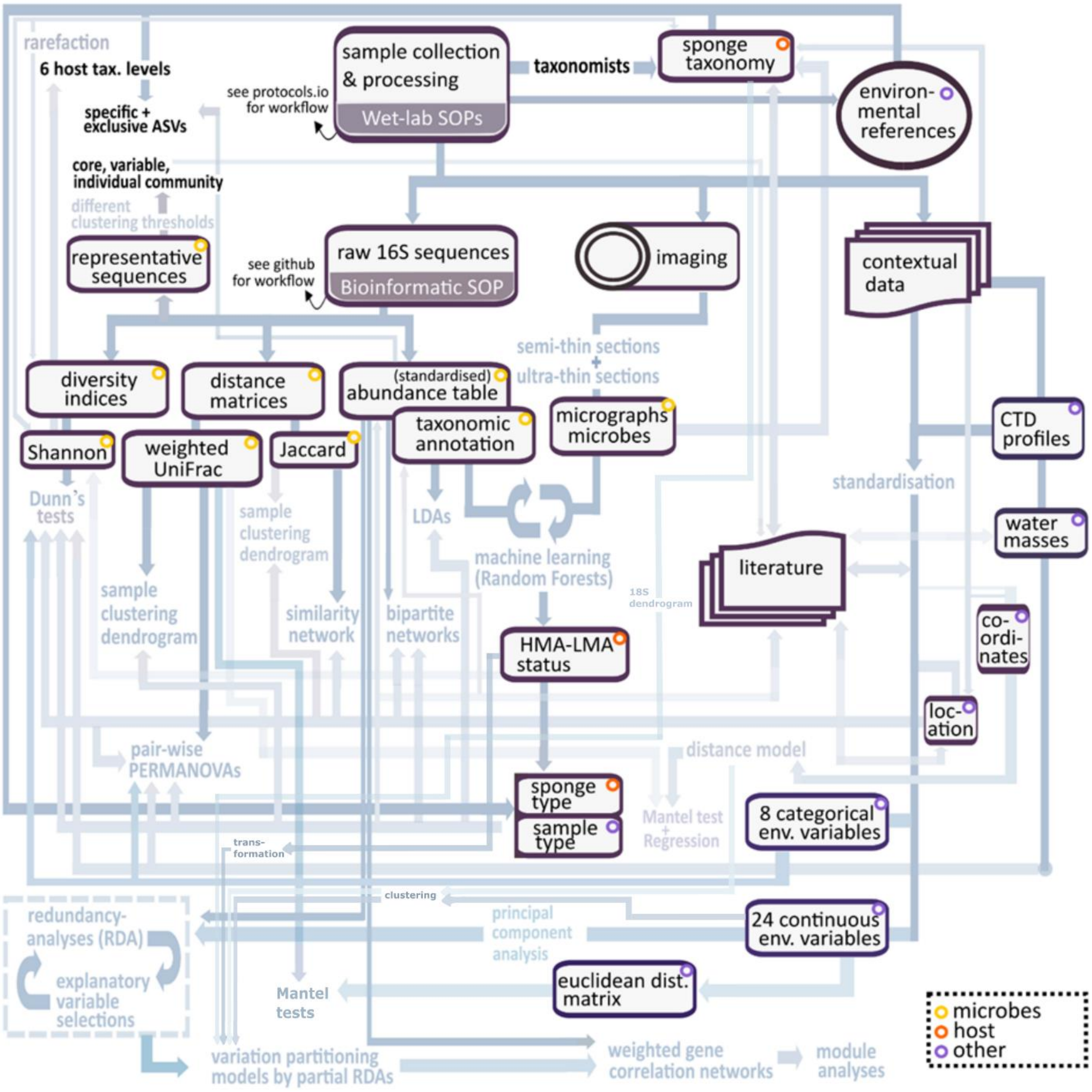

Supplementary Methods 1 The workflow used for statistical analyses of the presented data. Details on the standard operation procedures (SOPs) were deposited on protocols.io (wet-lab SOP) and github (bioinformatic processing of raw *16S* rRNA gene sequences).

Supplementary Methods 2

- **18S and COI sequencing**

Fragments of the *18S* rDNA (*18S*) gene were amplified for sponges using the primer pair SP18a F-SP18g R (SP18a F: 5′-CCTGCCAGTAGTCATATGCTT-3′; SP18g R: 5′-CCTTGTTACGACTTTTACTTCCTC-3′). The polymerase chain reaction (PCR) program for *18S* was 95 °C/2 min - (95 °C/20 s - 57 °C/45 s - 72 °C/30 s) x 30 cycles - 72 °C/3 min. For the amplification of a fragment of the cytochrome *c* oxidase subunit I (*COI*), we the primer pair dgLCO F- COXI R (dgLCO F: 5′-GGTCAACAAATCATAAAGAYATYGG-3′; COXI R: 5′-TGTTGRGGGAAAAARGTTAAATT-3′). The polymerase chain reaction (PCR) program for *COI* was 94 °C/5 min - (94 °C/30 s - 42 °C/1 min - 72 °C/30 s) x 30 cycles - 72 °C/7 min. Amplification of both *18S* and *COI* was performed in 10 µL reactions, using 0.05 µL of Taq DNA Polymerase, 1 µL Green Buffer, 0.2 µL dNTPs, 7.25 µL DEPC water, 0.5 µL of the 10 Mm forward or reverse primers, and 1 µL of DNA template. PCR products were verified by gel electrophoresis on 1% agarose. Sequencing was conducted with the primers mentioned above, using (dideoxy chain termination/cycle sequencing) on an ABI 3730XL DNA Analyser (Applied Biosystems, USA) by Eurofins Genomics.

- **Statistical analyses**

Various programs were used for statistical analyses, executed either within R or python from the unix terminal. We provide more details for the different statistical analyses in the following:

- **Dunn´s tests** with Bonferroni correction for p-values were run in order to stastistically evaluate differences in microbial alpha-diversity between different sample groups. These analyses were run with the *dunnTest*-function of the *FSA*-packages in R.

- In order to statistically test for differences in microbial beta-diversity, **PERMANOVA** group significance and pairwise tests were run simultaneously via the *beta-group-significance* method (non-parametric MANOVA;[25]) of the QIIME2 *diversity* plugin with 999 permutations.

- **Rarefaction curves** (number of observed ASVs per sample against number of sequences) were calculated with the *alpha-rarefaction*- function of the QIIME2 *diversity* plugin. 100 rarefaction depths were included between the minimal and maximal depth. 10 rarefied feature tables were computed at each step. In addition to the previously discussed rarefaction curves, also adapted rarefaction curves were created, showing microbial richness (number of observed ASVs) as a function of the number of observed sponge species. This analysis was done with the help of a custom designed R-script. For more details consider main manuscript text.

- **Linear Discriminative Analyses (LDAs)** to test for significant enrichment or depletion of microbial taxa in different sample groups were run in a conda environment based on the Linear Discriminant Analysis Effect Size (LEfSe) algorithm (version 1.0.0; [26]) and outputs subsequently processed further in R. The threshold on the absolute value of the logarithmic LDA score was set to 2, and the significance level alpha was set to 0.05.

- **Machine learning using the Random Forests algorithm** was conducted with the help of the *Scikit-learn* python package (version 0.17.1; [27]) in order to predict the HMA-LMA status of different sponge species. The procedure was done according to [1].

- A **principal component analysis (PCA)** was run based on the 24 continuous environmental parameters with the *PCA*-funtion of the *FactoMineR*-package in R.

- **Mantel tests** were run to assess correlations between environmental parameters (euclidean distances) and microbial community composition (weighted UniFrac distances) for the three sponge types. These analyses were conducted with the help of the *mantel*-function of the *vegan*-package in R, using Spearman-correlations and 999 permutations.

- In order to analyse distance-decay relationships of the deep-sea sponge microbiome, a **distance model** was set-up with the help of the *marmap*-package in R. A transition object was computed with a minimum depth constraint, preventing paths in water depths shallower than 200 m, then least cost distances were computed for the transition matrix. Mantel tests were performed with the help of the

mantel-function of the *vegan*-package in R in order to assess correlations between the dissimilarity of the microbial communities and the geographical distance. Regressions were also computed.

- With the help of **redundancy analyses (RDA)**, we determined the main environmental drivers of microbial community composition for the different sponge types. Forward selection procedures were performed on groups of factors with redundancy analysis models via the *rda*-function in the *vegan*-package of R. The best fitting models were selected using the Akaike Information Criterion. The significances of the models were assessed by 999 permutations and variation partitioning performed with the help of the *varpart*-function in the *vegan*-package of R.

- In order to rank different drivers of the microbial community composition (sponge status, geographic location, sponge taxonomy, and environmental parameters), **overall variation partitioning modelling** was performed including all factors. This was done for the variable sponge microbial community and only those ASVs occurring in more than 10 samples per group (HMA, LMA_demo, LMA_glass). For this analysis categorical variables were transformed into continuous variables with the following approach: (i) the HMA-LMA status was transformed into a binary format (1: HMA, 0:LMA); (ii) to evaluate the location effect in the overall model, the distance matrix between locations (taken from the previously described and performed distance-decay analysis) was processed with a cluster analysis. Resulting clusters were sorted as a spatial gradient (1: southernmost location cluster; 9: northernmost location cluster); (iii) to evaluate the effect of sponge phylogeny on variations in the microbial community composition, 18S sequences of one random representative individual per sponge order were aligned and a dendrogram was calculated in Geneious Prime (version 2020.0.5). Based on this dendrogram, individual sponge orders (and the underlying sponge individuals) were continuously numbered after the observed similarity gradient; (vi) to rank an overall environmental effect, similarity matrices based on all continuos environmental factors (except the location coordinates) were processed in a clustering approach. The resultung clusters were sorted after increasing depth (1:shallowest, 5:deepest).

A significance level of $\alpha = 0.05$ was applied for all statistical analyses.

- **Network analyses**

- Weighted gene correlation network analysis (WGCNA)

In order to identify specific microbial taxa related with the identified key environmental drivers (i.e. depth-related parameters, temperature-related parameters, salinity, as well as nutrient (N, P, Si) and oxygen concentrations), we run weighted gene correlation network analyses (WGCNA) for each sponge type (HMA, LMA demosponges, LMA glass sponges). Following the publication by [28], our main steps for the WGCNA anaylsis were the following ones: (i) Construct the network. Here we used the one-step network construction and module detection function *blockwiseModules* of the WGCNA package in R. The function was run based on a manual choice of the soft thresholding power β, after analysis of the network topology for various soft-thresholding powers. The equations are available in [28]. (ii) Identify modules (hierarchical clustering, Dynamic Tree Cut). (iii) Relate modules to environmental data. (iv) Study module relationships (Eigengene networks). (v) Find the key drivers in interesting modules (intramodular connectivity, causality testing). In order to extract only those microbes which had the strongest correlation with the other members of their module, and which had the strongest correlation with the examined key parameters (depth, temperature, salinity, nutrients/oxygen), we chose the cut-off value of 0.8. The results were then either visualised by heattrees or in a network format, with latter being constructed with the help of the Force Atlas 2 algorithm in Gephi.

- Similarity network and bipartite network analysis

In this study, bipartite network analyses were conducted for two main reasons: (i) to illustrate ASV distribution across sample types by a bipartite network between sponge + sample types ((HMA sponges, LMA_demo sponges, LMA_glass sponges, seawater, sediment)) and microbial taxa (ii) to assess connectivity of different sponge grounds by a bipartite network between sponge grounds (location) and microbial taxa.
In addition to bipartite networks, a similarity network was constructed between the analysed sponge ground locations based on microbial Jaccard distances. This was done as a complementary approach to the bipartite network in order to assess similiarity and connectivity of different sponge grounds.
As some readers may not be familiar to the measures of such network analyses, we have compiled an introduction/overview of some basic concepts in the following:

Betweenness centrality: Can be seen as a measure of the „bridging role" of a location in the network. A high betweenness centrality thus indicates locations within the network that are critical for the connectivity of all locations.
Module: A cluster of locations with similar microbial community compositions.
The module degree is helpful to identify each locations importance in driving connectivity between locations. A high **within-module degree** means that the respective location plays an important role for the connectivity between locations within the cluster. Whereas a high **between-module degree** implies that the respective location is relevant to maintain a connectivity between clusters.

Supplementary References

* literature marked with a blue asterisk served as reference for water mass identification

1. Moitinho-Silva, L. *et al.* Predicting the HMA-LMA status in marine sponges by machine learning. *Front. Microbiol.* **8,** 1–14 (2017).
2. Costello, M. J. *et al.* Marine biogeographic realms and species endemicity. *Nat. Commun.* **8,** 1–9 (2017).
3. Garcia, H. E. *et al.* World Ocean Atlas 2018. *NOAA Atlas NESDIS 83* **3,** (2018).
4. Garcia, H. E. *et al.* World Ocean Atlas 2018. *NOAA Atlas NESDIS 84* **4,** (2018).
5. Locarnini, R. A. *et al.* World Ocean Atlas 2018. *NOAA Atlas NESDIS 81* **1,** (2018).
6. Zweng, M. M. *et al.* World Ocean Atlas 2018. *NOAA Atlas NESDIS 82* **2,** (2018).
7. Olsen, A. *et al.* GLODAPv2.2019 - an update of GLODAPv2. *Earth Syst. Sci. Data* **11,** 1437–1461 (2019).
8. Olsen, A. *et al.* An updated version of the global interior ocean biogeochemical data product, GLODAPv2.2020. *Earth Syst. Sci. Data* **12,** 3653–3678 (2020).
9. NASA Goddard Space Flight Center, Ocean Ecology Laboratory, Ocean Biology Processing Group. Moderate-resolution Imaging Spectroradiometer (MODIS) Aqua Particulate Inorganic Carbon Data. doi:data/10.5067/AQUA/MODIS/L3M/PIC/2018 (2018).
10. NASA Goddard Space Flight Center, Ocean Ecology Laboratory, Ocean Biology Processing Group. Moderate-resolution Imaging Spectroradiometer (MODIS) Aqua Particulate Organic Carbon Data. doi:data/10.5067/AQUA/MODIS/L3M/POC/2018 (2018).
11. NASA Goddard Space Flight Center, Ocean Ecology Laboratory, Ocean Biology Processing Group. Moderate-resolution Imaging Spectroradiometer (MODIS) Aqua Chlorophyll Data. doi:data/10.5067/AQUA/MODIS/L3M/CHL/2018 (2018).
12. *Ridgway, N. M. Temperature and salinity of sea water at the ocean floor in the New Zealand region. *New Zeal. J. Mar. Freshw. Res.* **3,** 57–72 (1969).
13. *Loeng, H. Features of the physical oceanographic conditions of the Barents Sea. *Polar Res.* **10,** 5–18 (1991).
14. *Fahrbach, E., Rohardt, G. & Krause, G. The Antarctic coastal current in the southeastern Weddell Sea. *Polar Biol.* **12,** 171–182 (1992).
15. *Freiwald, A., Hühnerbach, V., Lindberg, B., Wilson, J. B. & Campbell, J. The Sula Reef Complex, Norwegian shelf. *Facies* **47,** 179–200 (2002).
16. *Lavin, L. *et al.* in *The Sea* 933-1001 (Harvard University Press, 2004).
17. *Hu, H., Liu, Q., Lin, X. & Liu, W. The South Pacific Subtropical Mode Water in the Tasman Sea. *J. Ocean Univ. China* **6,** 107–116 (2007).
18. *Aksenov, Y., Bacon, S., Coward, A. C. & Holliday, N. P. Polar outflow from the Arctic Ocean: A high resolution model study. *J. Mar. Syst.* **83,** 14–37 (2010).
19. *Rüggeberg, A., Flögel, S., Dullo, W. C., Hissmann, K. & Freiwald, A. Water mass characteristics and sill dynamics in a subpolar cold-water coral reef setting at Stjernsund, northern Norway. *Mar. Geol.* **282,** 5–12 (2011).
20. *Buhl-Mortensen, L. *et al.* in *Seafloor Geomorphology as Benthic Habitat* 703–715 (Elsevier, 2012).

21. *Chiswell, S. M., Bostock, H. C., Sutton, P. J. H. & Williams, M. J. Physical oceanography of the deep seas around New Zealand: A review. *New Zeal. J. Mar. Freshw. Res.* **49,** 286–317 (2015).

22. *Dever, M., Hebert, D., Greenan, B. J. W., Sheng, J. & Smith, P. C. Hydrography and coastal circulation along the Halifax Line and the connections with the Gulf of St. Lawrence. *Atmos.-Ocean* **54,** 199–217 (2016).

23. *Storesund, J. E. *et al.* Linking bacterial community structure to advection and environmental impact along a coast-fjord gradient of the Sognefjord, western Norway. *Prog. Oceanogr.* **159,** 13–30 (2017).

24. *Roberts, E. M. *et al.* Oceanographic setting and short-timescale environmental variability at an Arctic seamount sponge ground. *Deep Sea Res. Part I Oceanogr. Res. Pap.* 98–113 (2018).

25. Anderson, M. J. A new method for non-parametric multivariate analysis of variance. *Austral Ecol.* **26,** 32–46 (2001).

26. Segata, N. *et al.* Metagenomic biomarker discovery and explanation. *Genome Biol.* **12,** R60 (2011).

27. Pedregosa, F. *et al.* Scikit-learn: machine learning in python. *J. of Machine Learn. Res.* **12,** 2825–2830 (2011).

28. Langfelder, P. & Horvath, S. WGCNA: An R package for weighted correlation network analysis. *BMC Bioinformatics* **9,** 559 (2008).
