# Supplementary material for: Biodiversity, environmental drivers, and sustainability of the global deep-sea sponge microbiome
Source: Nat Commun. 2022 Sep 2;13:5160. doi: 10.1038/s41467-022-32684-4 (PMC9440067; doi:10.1038/s41467-022-32684-4)
Supplement: Supplementary file 3 — Reporting Summary [file 41467_2022_32684_MOESM3_ESM.pdf]

Corresponding author(s): Kathrin Busch
Ute Hentschel

Last updated by author(s): 22.07.2022

Reporting Summary

Nature Portfolio wishes to improve the reproducibility of the work that we publish. This form provides structure for consistency and transparency in reporting. For further information on Nature Portfolio policies, see our Editorial Policies and the Editorial Policy Checklist.

Statistics

For all statistical analyses, confirm that the following items are present in the figure legend, table legend, main text, or Methods section.

n/a	Confirmed	
☐	☒	The exact sample size (*n*) for each experimental group/condition, given as a discrete number and unit of measurement
☐	☒	A statement on whether measurements were taken from distinct samples or whether the same sample was measured repeatedly
☐	☒	The statistical test(s) used AND whether they are one- or two-sided<br>*Only common tests should be described solely by name; describe more complex techniques in the Methods section.*
☐	☒	A description of all covariates tested
☐	☒	A description of any assumptions or corrections, such as tests of normality and adjustment for multiple comparisons
☐	☒	A full description of the statistical parameters including central tendency (e.g. means) or other basic estimates (e.g. regression coefficient) AND variation (e.g. standard deviation) or associated estimates of uncertainty (e.g. confidence intervals)
☐	☒	For null hypothesis testing, the test statistic (e.g. *F*, *t*, *r*) with confidence intervals, effect sizes, degrees of freedom and *P* value noted<br>*Give P values as exact values whenever suitable.*
☒	☐	For Bayesian analysis, information on the choice of priors and Markov chain Monte Carlo settings
☐	☒	For hierarchical and complex designs, identification of the appropriate level for tests and full reporting of outcomes
☒	☐	Estimates of effect sizes (e.g. Cohen's *d*, Pearson's *r*), indicating how they were calculated

Our web collection on statistics for biologists contains articles on many of the points above.

Software and code

Policy information about availability of computer code

Data collection	No specific software was designed to collect data.
Data analysis	16S reads were processed within the QIIME2 environment (version 2019.10). Amplicon sequence variants (ASVs) were generated using the DADA2 algorithm. In order to taxonomically classify our 16S data, we used the SILVA ribosomal RNA gene database version (version 138 SSU Ref NR 99). Machine learning to predict the HMA-LMA status of different sponge species was conducted, following Moitinho-Silva et al. 2017 Front. Microbiol., with the help of the Scikit-learn python package (version 0.17.1). Linear Discriminative Analyses (LDAs) to test for significant enrichment or depletion of microbial taxa in different sample groups were run in a conda environment based on the Linear Discriminant Analysis Effect Size (LEfSe) algorithm (version 1.0.0). R (versions 3.0.2, 3.5.1, 3.6.2) was used for a large variety of data analyses: Weighted gene correlation network analyses (WGCNA) were done with the help of the "WGCNA" package (version 1.70-3). Dunn´s tests with Bonferroni corrections were run with the "FSA" package (version 0.9.3). A principal component analysis (PCA) was conducted based on continuous environmental parameters with the "FactoMineR" package (version 2.4). To analyse distance-decay relationships of the deep-sea sponge microbiome, a distance model was set-up with the help of the "marmap" package (version 1.0.5). Mantel tests & redundancy analyses were run with the "vegan" package (version 2.5-6). Besides statistical analyses, R (especially the "ggplot2" package; version 3.3.5) was also used for creating data visualisations. Additional programmes used for visualisation were Inkscape (version 0.92.4), python (version 3.7.3), Anvi'o (version 6.2), QGIS (version 3.4.4), Blender (version 2.92.0), and gephi (version 0.9.2). To evaluate the effects of sponge phylogeny on variations in the microbial community composition, 18S sequences were processed in Geneious Prime (version 2020.0.5).<br>The computational script of our core bioinformatic pipeline (incl. visualisations of technical validations) was deposited on GitHub: https://kathrinbusch.github.io/16S-AmpliconCorePipeline/.

For manuscripts utilizing custom algorithms or software that are central to the research but not yet described in published literature, software must be made available to editors and reviewers. We strongly encourage code deposition in a community repository (e.g. GitHub). See the Nature Portfolio guidelines for submitting code & software for further information.

Data

Policy information about availability of data

All manuscripts must include a data availability statement. This statement should provide the following information, where applicable:

- Accession codes, unique identifiers, or web links for publicly available datasets
- A description of any restrictions on data availability
- For clinical datasets or third party data, please ensure that the statement adheres to our policy

The raw sequence data generated in this study (16S, 18S, and COI) have been deposited within an Umbrella BioProject in the NCBI database under accession code PRJNA664762 [https://www.ncbi.nlm.nih.gov/bioproject/PRJNA664762]. SILVA data (version 138 SSU Ref NR 99) used to classify 16S amplicon sequences is available at https://www.arb-silva.de/. Important intermediate outputs of processed 16S data (i.e. ASV table and ASV taxonomy) were archived in the Zenodo database under accession code 10.5281/zenodo.6896034 [https://doi.org/10.5281/zenodo.6896034]. The ecological meta data and CTD profiles compiled in this study are available in the PANGAEA database under accession codes 10.1594/PANGAEA.923033 [https://doi.org/10.1594/PANGAEA.923033] and 10.1594/PANGAEA.923035 [https://doi.org/10.1594/PANGAEA.923035], respectively. In addition to our newly generated data, we used several publicly available resources to retrieve further data: the Word Ocean Atlas (version WOA18) [https://www.ncei.noaa.gov], GLODAP (version v2 2020) [https://www.ncei.noaa.gov], MODIS satellite data (NASA Goddard Space Flight Center 2018) [https://oceandata.sci.gsfc.nasa.gov/], the ETOPO1 1 Arc-Minute Global Relief Model (NOAA 2009) [https://www.ngdc.noaa.gov/mgg/global/], and the Sponge Microbiome Project dataset (Thomas et al. 2016: Supplementary Data 2 [https://doi.org/10.1038/ncomms11870]). For data standardisation Aphia IDs were retrieved from the World Register of Marine Species (WoRMS Editorial Board 2020) [https://www.marinespecies.org/]. Source data are provided with this paper. Other data supporting the findings of this study are available within the article and its Supplementary Information and Supplementary Data files.

Field-specific reporting

Please select the one below that is the best fit for your research. If you are not sure, read the appropriate sections before making your selection.

☐ Life sciences ☐ Behavioural & social sciences ☒ Ecological, evolutionary & environmental sciences

For a reference copy of the document with all sections, see nature.com/documents/nr-reporting-summary-flat.pdf

Ecological, evolutionary & environmental sciences study design

All studies must disclose on these points even when the disclosure is negative.

Study description	We present a large-scale analysis of microbial diversity in deep-sea sponges (Porifera) from scales of sponge individuals to ocean basins, covering 52 locations, 1077 host individuals including understudied glass sponges, and 469 reference samples, collected anew during 21 ship-based expeditions. We have generated 50 metadata entries for each sample, spanning geographic, biogeochemical, and physical parameters. The majority of samples were collected within the EU project SponGES (grant agreement no. 679849) in compliance with all rules and regulations of this grant agreement.
Research sample	For this study, we have sampled 169 sponge species, which cover 107 sponge genera, 52 families, 20 orders, and 4 classes. Our focus was on adult, apparently healthy sponge individuals. These samples were collected to analyse the associated microbial community composition and to determine the host taxonomic identification. As samples originate from 52 sponge ground locations from different oceans world-wide, we call this study the global Deep-sea Sponge Microbiome Project. For this large-scale dataset, we collected representative sponges at every sampling site. In addition to deep-sea sponges, we have also collected and analysed seawater and sediment samples as environmental reference.
Sampling strategy	Data presented in this paper were collected during 21 ship-based deep-sea expeditions, primarily in the North Atlantic but with representative samples from the Pacific, Arctic and Southern Oceans. Sampling was performed from onboard ship at sites of interest in transects (e.g. with ROV) and/or during point events (e.g. with CTD), depending on the used sampling equipment and aimed sample type (sponge, seawater, sediment). More details on event footprints can be found in the PANGAEA database [https://doi.org/10.1594/PANGAEA.923033 and https://doi.org/10.1594/PANGAEA.923035]. No sample size calculation was performed before, but it was aimed for three to four biological replicates per sponge species per site. As time is strongly limited during deep-sea dives and expeditions, three to four biological replicates was the highest feasible number. Similar numbers of biological replicates are also typically used for shallow-water sponge microbiome studies. Another aim during ship-based sampling was to gather as much ecological metadata as possible. Seawater was typically collected in three to four replicates of water (2L per replicate) at the same location where sponges were collected. Sediment was also collected in three to four biological replicates from the same location where sponges and water were collected.
Data collection	Strict standard operating procedures (SOPs) were established to reduce technical variation to a minimum. The detailled wet-lab standard operating procedure was archived at protocols.io [https://dx.doi.org/10.17504/protocols.io.kxygxer1kv8j/v1].<br>Details about the sampling cruises are listed in Supplementary Table 1. Cruise identifiers are the following ones: Hudson2016-019; PS96 (ANT XXXI/2); SO254; PS80 (ARK-XXVII/2); PS107 (ARK-XXXI/2); KB2017610; MLB2017001; GS2016109A; GS2017110; SPONGES 0617; HB2016952; CV13012; 0915S; HB27102017; JR17003a; PAA2014007; GS2018108; Azores2018; PS101 (ARK-XXX/3); MSM86; H045.<br>Sampling was conducted with the help of the following instruments: Bottle, Niskin (ROV); Bottle, Niskin (SHIP); Bottle, Niskin (SUB); Corer, Box (BC); Corer, Giant Box (GBC); Corer, Multi (MUC); Corer, Push (PUC_ROV); Corer, TV Multi (TVMUC); CTD/Rosette (CTD-RO); Dredge, anchor (DRG_A); Dredge, Chain Bag (DCB); Dredge, rock (DRG_R); Dredge, triangular (DRG_T); Grab (G); Mooring (long time), acoustic (MOORY); Remotely operated vehicle (ROV); Sediment net (ROV); Submersible, LULA1000 (SUB); Television-Grab (TVG); Trawl, Agassiz (AGT); Trawl, Alfredo (AT); Trawl, Beam (BEAM); Trawl, Bottom (BT); Trawl, Otter (OT).

	Details about the use of those instruments can be found at the PANGAEA database [https://doi.org/10.1594/PANGAEA.923033 and https://doi.org/10.1594/PANGAEA.923035].<br><br>Responsible for the sample collection were: Antje Boetius, Ana Colaço, Asimenia Gavriilidou, Ana Riesgo, Beate M. Slaby, Detmer Sipkema, Ellen Kenchington, Emyr Martyn Roberts, Furu Mienis, Hans Tore Rapp, Ingo Schewe, Javier Cristobo, Joana Xavier, Kathrin Busch, Lindsay Beazley, Luisa Federwisch, Melanie Bergmann, Marie Creemers, Pilar Ríos, Peter J. Schupp, Ruth Flerus, Sadie Mills, Thomas Soltwedel, Wolfgang Bach, and others.
Timing and spatial scale	This global collection effort spans distance ranges of 10 to 10,000 kms. The samples were collected between 2012-07-15 and 2019-09-17. The exact timing of sampling was dependent on ship availability for deep-sea expeditions. At least one cruise was conducted every year. The dates of the individual sampling cohorts are as follows:<br>2012-07-15 to 2012-07-30; 2013-08-20 to 2013-08-27; 2014-09-22 to 2014-10-19; 2015-07-16 to 2015-07-27; 2015-12-06 to 2016-02-14; 2016-06-18 to 2016-06-27; 2016-07-19 to 2016-08-16; 2016-09-08 to 2016-09-09; 2016-09-09 to 2016-10-23; 2017-01-27 to 2017-02-26; 2017-04-26 to 2017-05-02; 2017-06-11 to 2017-06-25; 2017-07-20 to 2017-08-06; 2017-07-23 to 2017-08-19; 2017-08-31 to 2017-09-07; 2017-10-27 to 2017-10-27; 2018-02-18 to 2018-03-12; 2018-05; 2018-07-04 to 2018-07-11; 2018-07-28 to 2018-08-14; 2019-08-14 to 2019-09-17.
Data exclusions	No data was excluded from the initial analysis. Several samples were dropped subsequently from the analysis during processing. Our filtering steps that led to dropping of samples included: (i) a removal of sponges with an ambiguous host taxonomic identification, (ii) a removal of contaminated samples (based on unrobust microbial fingerprints), and a removal of samples with less than 5000 reads (for more details on the bioinformatic filtering steps and quality criteria see https://kathrinbusch.github.io/16S-AmpliconCorePipeline/). Altogether, 1077 sponge individuals, 355 seawater samples and 114 sediment samples were collected and processed in a standardised way. Following removal of samples that did not pass our quality criteria, 931 sponges, 355 seawater samples, and 108 sediment samples (1394 samples in total) were included for subsequent analyses
Reproducibility	This study is a field study and does not involve any manipulative laboratory experiments. General measures taken to verify the reproducibility include the development and open sharing of standard operating procedures [https://dx.doi.org/10.17504/protocols.io.kxygxer1kv8j/v1 and https://kathrinbusch.github.io/16S-AmpliconCorePipeline/], and a high replication. Due to the high overall sample number, we are confident that the observed general patterns are robust.
Randomization	Sponge collection at each location was conducted in a randomized way: For each sponge species the first 1-4 sponge individuals encountered during the dive were collected.
Blinding	Samples were given sequential field codes with no indication of metadata (field notes were recorded). Sample processing was done by different persons at different steps (e.g. in the field, in the wet-lab, at the sequencing facility, at the computer). We treated all samples in a standardised way, and linked all notes and metadata other than sample type only at the very end.

Did the study involve field work? ☒ Yes ☐ No

Field work, collection and transport

Field conditions	As the focus of this study was the collection and evaluation of a large amount of metadata (more than 50 parameters), the generated information exceeds the available space of this text box. It can therefore be found in the PANGAEA database: https://doi.org/10.1594/PANGAEA.923033 and https://doi.org/10.1594/PANGAEA.923035.
Location	Samples were collected between 6 - 4833 m water depth.<br>Due to the large amount of samples we here report the spatial coverage, instead of individual sampling coordinates:<br>North-bound Latitude: 86.866 N; South-bound Latitude: -74.9927 N; East-bound Longitude: 179.8179 E; West-bound Longitude: -178.0261 E. Detailled information can be found in the PANGAEA database: https://doi.org/10.1594/PANGAEA.923033 and https://doi.org/10.1594/PANGAEA.923035.
Access & import/export	Samples for microbial molecular analyses were processed in a timely and sterile manner onboard ship, and transported back to the home laboratory frozen. In order to sample responsibly, to use precious deep-sea samples efficiently, and to integrate different data types (e.g. microbiome with sponge taxonomy), sponges were shared among several researcher onboard ship and subsequently processed by different experts.<br>Many samples of these deep-sea collections effort originate from international waters where sampling permits are not required. When permits were needed, they were compiled by the respective cruise PIs and can be made available upon request. Import of samples was done according to the relevant customs and transportation rules and regulations. Further details can be found in the SponGES grant agreement: 679849.
Disturbance	A preventive analysis was conducted prior to the project start in order to evaluate environmental risks and benefits (details can be found in the SponGES grant agreement: 679849). A possible concern is the disturbance of deep-sea life and ecosystems. However, samples were obtained with great caution in that frequently only sponge individuals were picked by sophisticated ROV technology that was operated by technology experts. For this reason, the impact on the environment was as negligible as can possibly be.

Reporting for specific materials, systems and methods

We require information from authors about some types of materials, experimental systems and methods used in many studies. Here, indicate whether each material, system or method listed is relevant to your study. If you are not sure if a list item applies to your research, read the appropriate section before selecting a response.

Materials & experimental systems

n/a	Involved in the study
☒	☐ Antibodies
☒	☐ Eukaryotic cell lines
☒	☐ Palaeontology and archaeology
☐	☒ Animals and other organisms
☒	☐ Human research participants
☒	☐ Clinical data
☒	☐ Dual use research of concern

Methods

n/a	Involved in the study
☒	☐ ChIP-seq
☒	☐ Flow cytometry
☒	☐ MRI-based neuroimaging

Animals and other organisms

Policy information about studies involving animals; ARRIVE guidelines recommended for reporting animal research

Laboratory animals	No laboratory animals were used.
Wild animals	The focus of this study was on adult, healthy sponge individuals belonging to 169 sponge species. Samples were flash-frozen immediately after collection for analyses of the associated microbial community composition. No manipulative experiments with wild animals were performed.
Field-collected samples	Samples of this study were collected in the field with the help of sophisticated deep-sea technology (see above). They were flash-frozen immediately after collection for analyses of the associated microbial community composition. Subsequent to sequencing, the samples (i.e. sponge tissue pieces, seawater filters, and sediments) were transferred into long-term storage at -80°C.
Ethics oversight	Research activities targeting sponges do not fall within the scope of application of neither the European nor any national relevant legislation on animal welfare, therefore they do not raise any ethical issues (more details can be found in the SponGES grant agreement: 679849).

Note that full information on the approval of the study protocol must also be provided in the manuscript.
