# Supplementary material for: Publisher Correction: Biodiversity, environmental drivers, and sustainability of the global deep-sea sponge microbiome
Source: Nat Commun. 2022 Nov 1;13:6546. doi: 10.1038/s41467-022-33966-7 (PMC9626464; doi:10.1038/s41467-022-33966-7)
Supplement: Supplementary file 1 — Description of Additional Supplementary Files [file 41467_2022_33966_MOESM1_ESM.pdf]

Description of Additional Supplementary Files

File Name: Supplementary Data 1
Description: Alphabetic listing of the 169 taxonomically identified deep-sea sponge species covered in this study. AphiaID refers to the official species identifier that is used in the World Register of Marine Species.

File Name: Supplementary Data 2
Description: List of 66 sponge species which harboured host species-exclusive ASVs. Species with ≥ 4 sponge individuals are marked in bold.

File Name: Supplementary Data 3
Description: Results of statistical testing (Dunn's tests) conducted to assess variations in microbial alphadiversity (Shannon index) between water masses for the different sample types. The tests were run two-sided and with Bonferroni corrections.

File Name: Supplementary Data 4
Description: Results of statistical testing (two-sided PERMANOVAs based on weighted UniFrac distances) conducted to assess differences in the microbial community composition between water masses for the different sample types.

File Name: Supplementary Data 5
Description: Results of statistical testing (twosided Mantel tests) conducted to assess correlations between environmental parameters (euclidean distances) and microbial community composition (weighted UniFrac dist ances) for the three sponge types. Group indicates to which category in the variation parititioning model each of the listed environmental parameters belongs. Parameters belonging to the same group are highly correlated with each other. Only those paramete rs which turned out to be the most relevant ones in the final variation partitioning models are shown.

File Name: Supplementary Data 6
Description: Results of statistical testing (twosided PERMANOVAs based on weighted UniFrac distances; 999 permutations) conducted to assess di fferences in the microbial community composition between sampling locations for the different sample types.

File Name: Supplementary Data 7
Description: Results of statistical testing (Dunn's tests) conducted to assess variations in microbial alpha diversity (Shannon index) bet ween sampling locations for the different sample types. were run two corrections.sided and with Bonferroni

File Name: Supplementary Data 8
Description: Results of statistical testing (twosided PERMANOVAs based on weighted UniFrac distances; 999 permutations) conductThe tests ed to assess differences in the microbial community composition between realms for the different sample types.

File Name: Supplementary Data 9

Description: Results of statistical testing (Dunn's tests) conducted to assess variations in microbial alpha diversity (Shannon index) between realms for the different sample types. The tests were run two sided and with Bonferroni corrections.

File Name: Supplementary Data 10
Description: Basic metadata for all samples of the dataset which passed the quality filtering steps.

File Name: Supplementary Data 11
Description: Dates of DNA extraction and names of sequencing runs for all samples of the dataset which passed the quality filtering steps.
